# Mobile Genetic Elements Associated with Antimicrobial Resistance Across One Health Interfaces in Africa: A Systematic Review and Meta-Analysis

**DOI:** 10.3390/antibiotics15050456

**Published:** 2026-04-30

**Authors:** Kedir A. Hassen, Jose Fafetine, Laurinda Augusto, Inacio Mandomando, Marcelino Garrine, Rogerio Marcos, Gudeta W. Sileshi

**Affiliations:** 1 Department of Bioscience and Public Health, Faculty of Veterinary (FAVET), Eduardo Mondlane University (UEM), Maputo 1102, Mozambique; jose.fafetine@uem.mz (J.F.); laurinda.augusto@uem.mz (L.A.); 2 Centre of Excellence in Agri-Food Systems and Nutrition (CE-AFSN), Eduardo Mondlane University (UEM), Maputo 1102, Mozambique; ceafsn@uem.mz; 3 College of Veterinary Medicine, Haramaya University, Dire Dawa P.O. Box 138, Ethiopia; 4 Centro de Investigação em Saúde de Manhiça (CISM), Maputo 1929, Mozambique; inacio.mandomando@manhica.net (I.M.); marcelino.garrine@manhica.net (M.G.); 5 Global Health and Tropical Medicine—GHTM, Associate Laboratory in Translation and Innovation Towards Global Health—LA-REAL, Instituto de Higiene e Medicina Tropical—IHMT, Universidade NOVA de Lisboa—UNL, 2829-516 Lisbon, Portugal; 6 Instituto Nacional de Saúde (INS), Maputo 1008, Mozambique; 7 Barcelona Institute for Global Health (ISGlobal), Carrer Rosselló 132E, 08036 Barcelona, Spain; 8 Department of Plant Biology and Biodiversity Management, College of Natural and Computational Sciences, Addis Ababa University (AAU), Addis Ababa P.O. Box 3434, Ethiopia; sileshigw@gmail.com

**Keywords:** antimicrobial resistance, mobile genetic elements, horizontal gene transfer, genomics, evidence synthesis, one health, Africa

## Abstract

**Background:** High infectious disease burden and uncontrolled antibiotic usage across human, animal, and environmental contaminants make antimicrobial resistance (AMR) a growing public health problem in Africa. Mobile genetic elements (MGEs) such plasmids, transposons, integrons, conjugative elements, and phages help spread AMR via horizontal gene transfer (HGT) across human, animal, food, and environmental sources. Despite growing evidence for antibiotic resistance genes (ARGs), Africa lacks a one-health-focused synthesis of mobile genetic element-mediated AMR. **Objective**: This systematic review and meta-analysis aimed to consolidate information on MGEs and ARGs in AMR dissemination throughout Africa’s one health interface. **Methods:** The literature was searched using PubMed, Scopus, and ScienceDirect. Observational. molecular epidemiology, whole genome sequencing (WGS), and metagenomic investigations of MGE-associated AMR in Africa were eligible. The study selection, data extraction, and quality assessment were performed by two independent reviewer and quality was graded using ROBVIS 2 utilizing Rayyan software. Narrative synthesis, random-effect meta-analysis, subgroup analysis, and meta-regression were utilized. **Results:** A total of 109 studies were included, with 91 studies contributing to the meta-analysis. MGEs reported were plasmids (71.7%) and integrons (54.8%). ARGs carried by MGEs were *bla*CTMX-M-15 (78.6%), *Sul*2 (69.6%), *bla*TEM (59.1%), and *tet*A (49.9%). Horizontal gene transfer was seen in 259 instances; however, transmission was unclear. In 442 observations, transmission pathways across human, animal, and environmental interfaces showed AMR prevalence of 75.1% in human, 98.0% in human–animal, and 61.3% in one health interface. Whole-genome sequencing was the most frequently used method for detecting MGEsThe pooled pathogen and AMR prevalence rates were 73.3% (95% CI: 60.5–83.7%) and 94% (95% CI: 85–98%), with significant heterogeneity (I^2^ = 97.8% and 97.4%, respectively). The prevalence of *Escherichia coli* was 93% and *Salmonella enterica* 85% in subgroup analysis. Fluoroquinolones, aminoglycosides, and beta-lactams were prevalent in humans (89.7%) and human–animal interactions (98.0%) according to AMR Class. **Conclusions:** Horizontal gene transfer has propagated MGE-mediated antimicrobial resistance across human, animal, and environmental interfaces in Africa. To combat AMR in Africa, coordinated, genomics-informed One Health surveillance and antibiotic stewardship are needed. Due to variability and publication bias, these data should be considered cautiously. Pooled data may only show descriptive patterns, and not necessarily precise continent-wide prevalence estimates.

## 1. Introduction

Antimicrobial resistance is globally acknowledged as a significant threat to public health, food security, and socioeconomic stability [1,2,3,4,5]. The World Health Organization strategy for antimicrobial resistance containment, published in 2001, established a framework of measures to prevent the increasing prevalence of antimicrobial-resistant microbes. In the European Union (EU), it is estimated that antimicrobial resistance pathogens are responsible for approximately 33,000 deaths per year and 4.95 million deaths globally, with health care expenditure and productivity losses amounting to EUR 1.5 billion every year [5,6,7,8]. The emerging consensus is that AMR is a big problem for the economy as well as for health care. The World Bank estimates that, by 2050, AMR will cause more than 10 million deaths per year, with a loss of more than 100 trillion dollars and a reduction of 2 to 5% in gross domestic product worldwide, especially in low- and middle-income countries (LMICs) [9]. The effect goes beyond health care and affects farming, food systems, and the environment, which shows how important it is for countries to work together [4,5,8].

Structural health care restrictions, insufficient diagnostic capability, limited antimicrobial stewardship, and an inadequate surveillance system are likely to put an unfair burden on LMICs [4,5,8]. Africa is one of the most vulnerable places in the world [4]. A high burden of infectious disease, rising use of antimicrobials in human and veterinary medicine, careless pharmaceutical regulation, and deficiency in water hygiene and sanitation infrastructure collectively establish conducive conditions for both emergency and sustained transmission of resistant pathogens [8,9]. International organizations, such as the WHO, FAO, and WOAH, stress that a One Health approach is essential for tackling AMR. This is because they recognize the interconnectedness of humans, animals, food systems, and environmental factors [4,5,8,9,10].

Mobile genetic elements are essential for the evolution and dissemination of antimicrobial resistance through horizontal gene transfer, enabling the transfer of resistance genes among bacterial species, hosts, and environments. MGEs facilitate rapid gene transfer, accelerating the emergence of multidrug-resistant pathogens, in contrast to vertical inheritance [11,12]. Horizontal gene transfer (HGT) is an important biological process that allows genes to be shared between different species. For example, it lets bacteria share genes that make them resistant to antibiotics [11,12]. Horizontal gene transfer primarily occurs through transformation, conjugation, and transduction, mainly facilitated by mobile genetic elements (MGEs) such as plasmids, transposons, integrons, integrative and conjugative elements, and bacteriophages [11,12]. These mobile genetic elements (MGEs) can facilitate the transfer of resistance genes within and between different bacterial species. This speeds up the spread of multidrug-resistant and extensively drug-resistant diseases [13,14].

Recent advancements in whole genome sequencing (WGS) and metagenomics have markedly enhanced the ability to differentiate ARGs, MGEs, and their genetic context with high resolution [11,12]. Genomic methods provide enhanced sensitivity and enable more accurate monitoring of resistance transmission pathways compared to conventional phenotypic methods [15,16]. However, the availability of sequence infrastructure and bioinformatic capacity is limited in most African countries, resulting in insignificant geographic and methodological variation in AMR surveillance and reporting [17,18].

AMR is a highly interconnected ecological system that links clinical settings, livestock production systems, food chains, wastewater environments, and natural ecosystems [17,18]. MGEs act as molecular channels that help spread AMR across the sector [18,19,20]. While mobile genetic elements (MGEs) are frequently reported in association with AMR, existing evidence is fragmented across different countries, bacterial species, and study designs. In addition, variation in methodology and reporting limit comparability across studies and complicate the effort to draw conclusions regarding the distribution and potential movements of resistance genes within a One Health framework.

This work aimed to address these gaps through a systematic review and meta-analysis of the available evidence on the occurrence and distribution of MGEs associated with AMR across the one health interface in Africa. The specific objective of this study was to (i) provide a synthesis of reported MGES types and ARGs associated with AMR and (ii) describe the pattern of occurrence in human, animal, and environmental settings. (iii) Provide a pooled descriptive estimate of AMR and pathogen prevalence. The finding is intended to support improved understanding of AMR patterns and inform future research and surveillance strategies, while acknowledging the heterogeneity and limitations of available evidence.

## 2. Results

### 2.1. Characteristics of Included Studies

**Figure 1:**
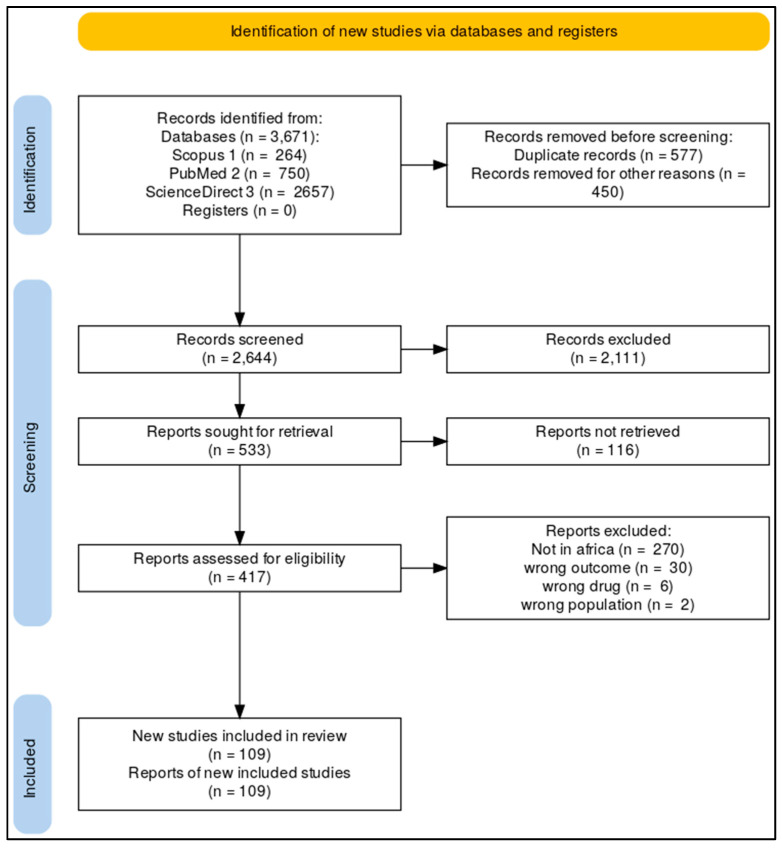
PRISMA flow diagram.

The quantitative and qualitative synthesis was included 109 published eligible articles. Supplementary Material S1 had the PRISMA checklist guidelines, and the PRISMA 2020 flow diagram [21] in Figure 1 shows how the selection process worked across databases, registers, and other sources.

**Figure 2:**
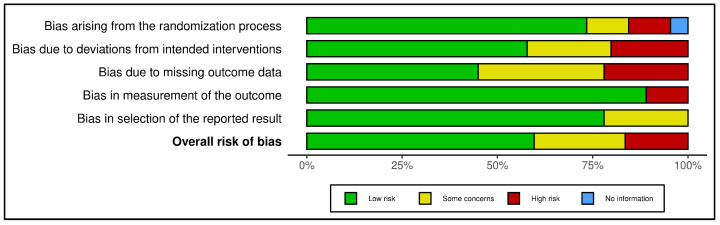
Summary of risk of bias assessment.

Supplementary Figure S1 provides a comprehensive report on the risk of bias across studies, including detailed assessments at the study level. Figure 2 shows that most domains were thought to have a low risk of bias. The outcome measurement and selection of the reported results had the highest percentages of low risk. However, notable concerns persist regarding deviations from intended interventions and missing outcome data, as higher proportions of certain concerns and high risk were identified in these areas. The overall assessment suggests a generally acceptable level of methodological quality, even though some studies still show moderate to high risks of bias in certain areas. Figure 2 illustrates the summary of bias risk across the studies.

### 2.2. MGEs Associated with AMR in Africa

Plasmids were the most commonly reported mobile genetic elements (MGEs) linked to antimicrobial resistance (AMR), succeeded by integrons and combinations that included insertion sequences (IS) in the observations presented (Table 1). Plasmids had the highest reporting frequency and a mean AMR prevalence of 71.7%, which is pretty high. Combinations that included insertion sequences were also often linked to high AMR prevalence, which was often over 80% (Table 1).

**Figure 3:**
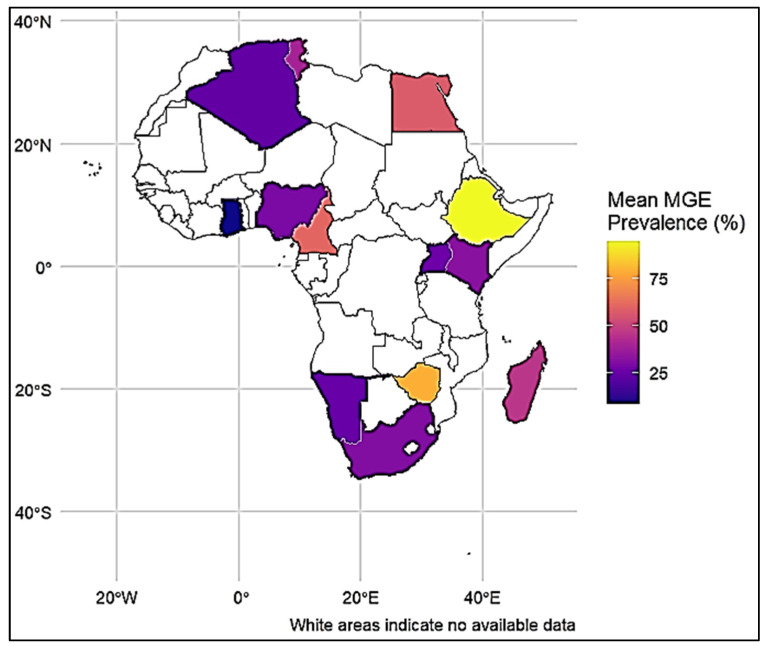
Geographical distribution of MGE-associated AMR in Africa.

Geographical mapping indicated disparities in MGE-associated AMR across African regions, with comparatively elevated values reported in East and Southern Africa relative to Central, West, and North Africa (Figure 3).

### 2.3. Distribution of ARGs Associated with MGEs

*Sul*2, *bla*CTX-M-15, and *tet*A were the most common antimicrobial resistance genes (ARGs) found in studies of MGEs (Table 2). The gene *bla*CTX-M-15 had the highest average prevalence among MGE-positive observations (78.6%), followed by the sulfonamide resistance genes (*sul1–3*) (69.6%). Other genes that were often reported were *bla*TEM and *qnrB*.

**Figure 4:**
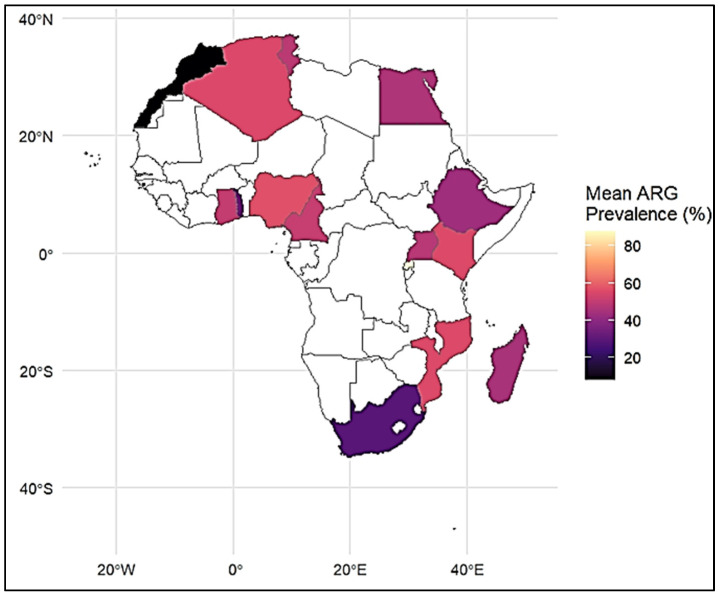
Geographical distribution of ARGs in Africa: country-level mean prevalence.

Maps of the spatial distribution of ARGs showed that the number of reports varied by region, with North, East, and Southern Africa having more reports than other regions (Figure 4).

### 2.4. Antimicrobial Classes Associated with MGEs

**Figure 5:**
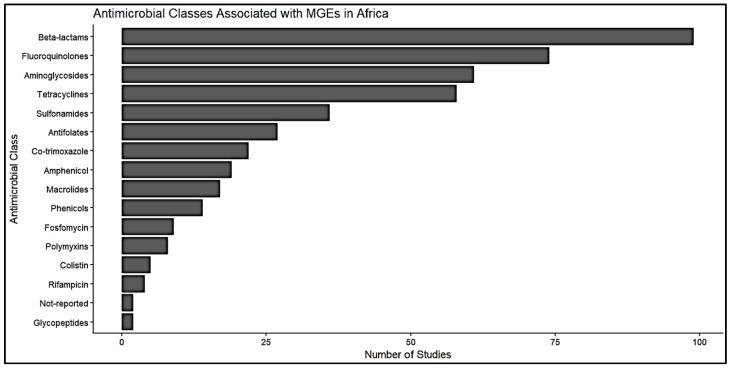
Antimicrobial Classes Associated with Mobile Genetic Elements in Africa.

The review identified sixteen antimicrobial classes; beta-lactams were the most frequently reported class (*n* = 99 observations), exhibiting a mean AMR prevalence of 64.2% among MGE-associated datasets (Figure 5). Fluoroquinolones, aminoglycosides, and tetracyclines were frequently documented. Some classes of antimicrobial drugs, like aminoglycosides and sulfonamides, had mean prevalence values that were pretty high (>80%) (Figure 5).

### 2.5. Distribution of AMR and MGEs Across One Health Interfaces

The human interface was the most common place for AMR and MGEs to happen (*n* = 134 observations). It had the highest average AMR prevalence (75.1%) and a high average MGE prevalence (47.8%) (Table 3). The human–animal interface had a very high mean prevalence of AMR (98.9%), while the human–environment interface had the highest mean prevalence of MGE (86.0%). But only a few observations (*n* = 12) found it. The animal–environment interface was the second most common (*n* = 91 observations), with AMR (54.5%) and MGE (32.8%) being the most common. There were 68 observations that mentioned One Health interfaces, with AMR at 61.3% and MGE at 40.2% (Table 3). Supplementary Figure S2, shows the comparative distribution of antimicrobial resistance (AMR) prevalence across one health domains: human, animal, and environmental sectors.

Evidence characterized as “cross-spread” among interfaces has been documented in multiple studies (Table 4). Nonetheless, in the majority of instances, this was deduced from the co-occurrence of MGEs and ARGs rather than through direct evidence of transmission pathways. Reported mechanisms encompassed horizontal gene transfer (HGT) and clonal dissemination, although directionality and causality were infrequently determined. 

Analysis of the distribution of ARGs across different One Health interfaces revealed distinct patterns for key resistance determinants. The distribution of antimicrobial resistance genes by the One Health Interface (study observations) is shown in Table 5.

### 2.6. Methods Used for Identifying MGEs

The primary technique employed for the identification of MGEs in the analyzed observations (*n* = 160) was WGS (Table 6). WGS had the highest MGE detection rate (100%), followed by genomic and bioinformatics methods (98.9%; *n* = 95 observations). Traditional methods, such as phenotypic characterization (*n* = 121 observations) and molecular detection and typing (*n* = 116 observations), demonstrated slightly lower but still high MGE detection rates (86.8% and 87.9%, respectively).

Genomic and metagenomic techniques were utilized in 160 observations, resulting in a marginally elevated mean prevalence of mobile genetic elements (38.8%) compared to conventional/PCR-based methods (36.2%; *n* = 333 observations) (Table 7).

### 2.7. Regional Variation in MGEs and ARGs

Using data from 461 observations, we looked at the frequency and prevalence of MGEs by region. We found that the prevalence of MGEs and AMR varied by region (Table 8). The average MGE prevalence was highest in North Africa (44.1%), then in East Africa (35.2%), West Africa (32.6%), and southern Africa (32.4%). We could not figure out how common MGEs were in Central Africa because there were not enough data. The average MGE prevalence and the average AMR prevalence in the regions were related in a positive but not linear way. West Africa had the highest overall AMR prevalence (77.4%), but its MGE prevalence was only moderate (32.6%) (Table 8).

There were significant differences between regions in the types of MGE that were found (Supplementary Figure S3). Plasmids were the most frequently recorded type of mobile genetic element in all regions. North Africa demonstrated considerable diversity and frequency of plasmid and integron documentation. East Africa reported complex combinations of MGEs, particularly a plasmid-insertion sequence with an extraordinarily high mean prevalence of 94.7%. Studies in Southern Africa often report integrons. There is a lack of information on certain types of MGEs from Central Africa. Supplementary Figure S3 shows how MGE types are spread out across Africa using heatmaps.

**Figure 6:**
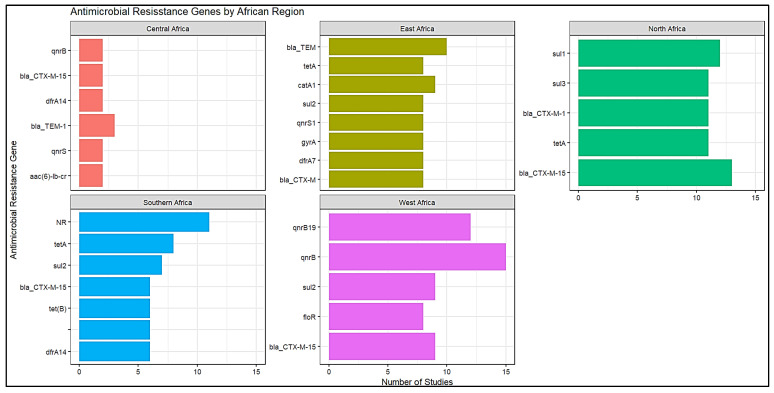
Antimicrobial resistance genes (ARGs) by African region.

A total of 500 distinct ARGs region combinations were documented, and the leading ARGs for each region are summarized in Figure 6. The *bla*CTX-M-15 gene was a prevalent antimicrobial resistance gene in North Africa, with a mean prevalence of 91.2%, and it was also documented in Central Africa (Figure 6). In West Africa, *qnr* genes (*qnr*B and *qnr*B19) exhibited significant prevalence. The gene *bla*TEM was commonly observed in East, West, and Southern Africa, while the genes *sul1* and *tet*A were prevalent in North Africa, as shown in Figure 6.

A Kruskal–Wallis test revealed no statistically significant difference in the prevalence of MGE among the studied regions (χ^2^ = 2.08, df = 3, *p* = 0.555). A country-specific analysis delineated critical research areas and significant prevalence rates. In Ethiopia, the average rate of MGEs was 94.7%. Ghana had the highest average AMR rate (98.9%) and the lowest MGE rate (8.5%). Egypt (in North Africa) and Nigeria (in West Africa) were the two countries that received the most attention.

### 2.8. Meta-Analysis of Pathogen and AMR Prevalence

We did a random-effects meta-analysis on 91 studies that looked at the overall prevalence of the target pathogens. These studies had 24,947 positive isolates and 12,323 resistance isolates. The overall prevalence of pathogens in Africa was 73.7% (CI: 60.5–83.7%), but there was a lot of variation between studies (I^2^ = 97.8%, τ^2^ = 2.03; Q = 4106.97, *p* < 0.001). Supplementary Figure S4, Forest plot shows pooled estimate of pathogen prevalence, estimates of effect sizes and their 95% confidence intervals (CI) across included studies.

There were 79 studies in the subgroup analysis by bacterial pathogens, with 23,178 positive isolates and 11,265 resistance isolates. The results showed that the outcome was much more common among bacterial pathogens, with a pooled estimate of 91.5% (CI: 79.7–96.7%) (Table 9). There was also a lot of variation between the studies (I^2^ = 97.7%). *Escherichia coli* had a higher overall prevalence (93.2%) than *Salmonella enterica* (84.5%) when looking at the subgroups by pathogen species (Table 9). There was also a lot of variation within the subgroups (I^2^ ≥ 97%) (Table 9). The analysis for differences between subgroups was not statistically significant (Q = 0.67, *p* = 0.4148). Supplementary Figure S4b, shows Forest plot of subgroup analysis for pathogen prevalence.

The subgroup analysis by antimicrobial class consisted of AMR prevalence reported in 91 studies with a total of 24,947 positive isolates and 12,323 resistance isolates. The random-effects logistic regression model estimated the AMR prevalence at 94.2% (CI: 0.855–0.978), indicating significant between-study heterogeneity (I^2^ = 97.4%; tau^2^ = 19.29; Q = 3494.56, *p* < 0.001). Details of Forest plot illustrating the overall pooled prevalence of AMR analysis are summarized in Supplementary Figure S4c. The pooled resistance estimates range from 92.7% for beta-lactams to 99.6% for tetracyclines (Table 10). The Forest plot of subgroup analysis for AMR prevalence. was not statistically significant (Supplementary Figure S4d).

Results of the subgroup analysis by geographic region are summarized in Table 11. The aggregated proportion of MGE-associated AMR was 94% (CI: 0.85 to 0.98) with high heterogeneity (I^2^ = 97.4%). The greatest proportion was noted in studies from the West (99.6; CI: 0.877–1.000), and the least was found in studies from the Central region (60.3; CI: 0.220–0.891) (Table 11). The forest plot showing regional subgroup prevalence estimates not statistical significance (Supplementary Figure S4e).

The estimated pooled pathogen prevalence at One Health interfaces was 82.7%. There are big differences, especially at the human (89.7%) and human–animal (98.0%) interfaces. The numbers for the animal-only (75.5%) and animal–environment interfaces (66.2%) were also very high. With a prevalence of 77.5%, the environment was also an important source. A One Health-integrated research study revealed a significant prevalence of 78.9%, accompanied by considerable heterogeneity (I^2^ = 97.8%). Supplementary Figure S4f shows the forest plot comparing prevalence across one health interfaces (meta-interface comparisons).

There was a lot of difference between the studies that were included. The I^2^ statistic, which measures the percentage of variability in prevalence estimates caused by heterogeneity rather than chance, was very high at 97.8%. This means that almost all of the variability seen between studies is likely due to real differences rather than random error. The between-study variance (τ^2^) was 2.03, which means that the true effect sizes varied a lot from study to study. Cochran’s Q statistic was also 4106.97 (*p* < 0.001), which showed that there was a lot of variation.

The funnel plot shows that the data is not symmetrical, which could mean that there is a publication bias. We used Egger’s linear regression test for funnel plot asymmetry to check this statistically. The test produced a t-value of 3.69 (df = 89) and a *p*-value of 0.0004, along with a bias estimate of 3.79 (SE = 1.03). Supplementary Figure S4g, shows funnel plot assessing potential publication bias among included studies.

The meta-regression revealed a correlation between AMR and MGE prevalence. Even after changing the study interface, design, laboratory methods, geographic location, and publication year, there was still a lot of unexplained variation (τ^2^ = 2.62; I^2^ = 96.8%), with no variation explained (R^2^ = 0.00%). A test for residual heterogeneity (QE = 1474.32, *p* < 0.001) showed that there were significant unexplained sources of variation. The overall moderation test (F = 1.60, *p* = 0.058) was close to being significant. The study design was a strong predictor, with cross-sectional studies linked to higher AMR/MGE rates (estimate = 7.01, *p* = 0.042). Table 12 shows that laboratory methods affected prevalence; for example, phenotypic characterization was linked to lower estimates (estimate = −2.35, *p* = 0.024).

Temporal evaluations revealed no substantial variation in the prevalence of MGEs and ARGs. Linear regression revealed some trends over time (β = −0.99 per year, *p* = 0.25; R^2^ = 0.015). Supplementary Figure S4h shows the temporal trend analysis of MGE prevalence over time. Supplementary Figure S4i shows the temporal trend of antibiotic resistance gene (ARG) prevalence. Supplementary Figure S4j shows the trends in AMR prevalence across One Health interfaces over time. Supplementary Figure S4k shows the quality-weighted temporal trend analysis of AMR prevalence, accounting for study quality and heterogeneity.

## 3. Discussion

### 3.1. MGEs Associated with AMR in Africa

The findings reported that plasmids, integrons, and insertion sequences and associated combinations are the facilitators of resistance spread, and this is in agreement with worldwide reports [22,23]. The indication for plasmids observed in this review is consistent with patterns reported across Europe, Asia, and the Americas, in addition to the worldwide dissemination of epidemic plasmid families such as *IncF*, *IncI*, *IncX*, and *IncA/C* [24,25]. This observation is pertinent to the international report emphasizing the crucial function of plasmids in horizontal gene transfer, a process that facilitates the swift spread of resistance genes among bacterial populations [26,27,28]. Integrons and plasmid–integron combinations demonstrated substantial contributions to antimicrobial resistance (AMR), corroborating global findings that class 1 integrons play a significant role in promoting multidrug resistance [15,29,30].

Nevertheless, their reduced reporting rate of MGEs in Africa probably shows methodological limitations and scarce application of genomic methods [31,32]. Insertion sequence associated MGEs, although rarely described, revealed a very high occurrence of AMR [33,34,35]. This evidence is relevant to global reports regarding their role in the mobilization of resistance genes, showing a more significant impact in African contexts [36,37,38,39], apparently due to substantial antimicrobial selection burden and scarce infection prevention infrastructure [40,41,42]. Geographical differences across African regions, with higher reported results in East and Southern Africa, probably show variances in surveillance intensity, antimicrobial usage, and study design rather than accurate epidemiological differences. Generally, these findings highlight the crucial necessity to strengthen genomic surveillance to better reveal the diversity and epidemiological significance of MGEs, within a One Health framework across the African continent [43,44,45].

### 3.2. ARGs Associated with MGEs in Africa

The findings reported *sul*2, *bla*CTX-M-15, and *tet*A dominate among MGE-associated resistance genes in Africa, these directly reflecting global antimicrobial resistance patterns [46,47,48], particularly in low- and middle-income regions [48,49]. The remarkable occurrence of *bla*CTX-M-15 among MGE-positive isolates has led to its designation as the most prevalent extended-spectrum β-lactamase globally [50,51,52]. This is mainly driven by its strong association with conjugative plasmids and transposable elements, in addition to its critical part in conferring resistance to third-generation cephalosporins in both hospital and community settings [52,53,54]. The high occurrence reported in African continent is relevant with worldwide genomic surveillance and probably shows widespread β-lactam use, limited diagnostic capacity, and efficient horizontal gene transfer [55,56,57].

The significant occurrence of sulfonamide (*sul1–3*) and tetracycline (*tetA*) resistance genes shows a persistent selective burden from previously and currently used antimicrobials [58,59,60,61], it is consistent with global reports where these genes are commonly positioned on plasmids and class 1 integrons across human, animal, and environmental sources [62,63,64]. The persistent exposure of other resistance genes, as well as *bla*TEM, *dfrA14*, and *qnrB*, highlights the accumulation of multidrug resistance genes. Geographical inconsistency, with higher reporting in North, East, and Southern Africa, probable shows variances in surveillance capacity, antimicrobial usage patterns, and research focus rather than true epidemiological difference. Overall, these findings align with global trends, where a few highly mobile and successful ARGs, particularly *bla*CTX-M-15 and *sul* genes, drive the AMR burden through MGE-mediated spread [62,63,64]. This emphasizes the importance of integrated monitoring approaches within a One Health framework to identify and control the transmission of resistance genes.

### 3.3. Antimicrobial Classes Associated with MGEs in Africa

The distribution of antimicrobial classes associated with MGEs indicates that β-lactams are reported as the most dominant, with a high AMR occurrence, relevant to the worldwide distribution of resistance mediated by extended-spectrum beta-lactamases [65,66,67]. The common association of fluoroquinolones, aminoglycosides, and tetracyclines additionally shows widespread multidrug resistance patterns driven by horizontal gene transfer [68,69,70,71]. This suggests that multidrug resistance genes are strengthening on plasmids and integrons [72,73,74], often at levels equal to or higher than what is reported around the globe, particularly in environmental and agricultural sources [75,76,77]. The determined resistance to classical antimicrobials highlights the sustained dependence on these agents in both human and animal sectors in Africa, in contrast to the decreasing trends in high-income countries [78,79,80]. Higher prevalence estimates observed for some antimicrobial classes should be interpreted with caution, as they may be due to study design, selective reporting, and limited observation.

### 3.4. Distribution Across One Health Interfaces in Africa

Across One Health interfaces, the human sector presented the highest reporting and AMR burden, while the human–animal interface exhibited extremely high AMR occurrence [81,82,83], highlighting probable transmission burden at this interface [84,85]. The high MGE occurrence in the human–environment interface suggests that environmental sources play a vital role in maintaining and disseminating resistance genes [86,87,88]. There are fewer studies, but the high occurrence of MGEs at the human–environment interface is consistent with global findings that wastewater and other environmental compartments act as important sources for MGE-mediated ARGs and [89,90,91]. Indication of cross-spread across interfaces was commonly inferred through co-occurrence of MGEs and ARGs, with horizontal gene transfer and clonal spread as mechanisms, even though direct transmission pathways remain poorly reported [92,93,94].

### 3.5. Methods Used for Identifying MGEs

Methodologically, whole-genome sequencing confirmed a higher detection rate for MGEs, highlighting the importance of genomic approaches for accurate AMR surveillance [89,90,91]. Genomic and bioinformatic methods also showed high detection rates. This highlights how advances in sequencing technologies and analytical processes improve the identification of plasmids, integrons, and other mobile elements, compared to older methods [14,95,96]. Even though conventional methods such as phenotypic characterization and molecular typing remained widely used and presented comparatively high detection rates, they may limit the identification of novel or complex MGEs, possibly underestimating the true diversity of resistance mechanisms [39,97,98]. The higher mean MGEs occurrence observed in genomic and metagenomic studies compared with PCR-based methods probably reflects improved sensitivity and broader detection capability instead of accurate epidemiological variances [99,100,101]. Collectively, these findings reported an ongoing methodological alteration to genome-based surveillance, suggesting that combining advanced genomic tools with conventional laboratory methods may deliver the most comprehensive framework for monitoring MGEs associated with AMR transmission, mainly within One Health surveillance systems [99,100,101].

### 3.6. Insights from Meta-Analysis: Pathogen and AMR Prevalence

The meta-analysis reported a very high pooled occurrence of AMR and pathogens, with significant heterogeneity, demonstrating significant variability across studies. This finding surpasses the predictions made in global assessments of low- and middle-income areas [99,100,101]. The prevalence of high resistance across various antimicrobial classes and interfaces amplifies the magnitude of the AMR challenge in Africa [102,103,104]. *Escherichia coli* exhibited a greater pooled occurrence relative to *Salmonella enterica*, which is consistent with its global incidence as a significant reservoir and efficient disseminator of resistance [105,106]. The absence of notable subgroup effects suggests that the threat posed by antimicrobial resistance in Africa is primarily shaped by ecological factors and the burden of antibiotic usage, rather than pathogen-specific determinants [107,108].

The occurrence of antimicrobial resistance (AMR) among recognized pathogens was significantly high, relevant to global reports that mobile genetic elements (MGEs) facilitate nearly universal multidrug resistance through the co-localization of resistance genes on plasmids and integrons [109,110]. Resistance patterns for β-lactams, fluoroquinolones, aminoglycosides, sulfonamides, and tetracyclines reflected global trends [111,112]; however, the continuing high resistance to older antibiotics certainly contrasts with the falling patterns observed in various high-income regions, demonstrating a sustained dependence on these medications in African continents [113,114,115].

Interface investigations confirmed that human and human–animal environments help as substantial transmission hotspots, maintained by significant environmental sources [116,117,118], relevant with the global One Health report [119,120,121]. Even though there are still differences and signs of publication bias [122,123,124], the fact that the findings are consistent across subgroups strengthens the strong conclusion that antimicrobial resistance (AMR) in Africa is widespread [125,126,127], ecologically complex, and best dealt with through integrated One Health surveillance and genomics-informed approaches [127]. However, significant heterogeneity, publication bias, and limited explanatory power of meta-regression models suggest that underlying drivers of antimicrobial resistance in Africa remain incompletely understood, particularly in relation to environmental factors and their interactions with human health. Overall, these findings align with global trends and emphasize the necessity of integrated surveillance and control strategies within a One Health framework.

### 3.7. Policy Implications

These findings reveal the importance of harmonized, one-health-oriented antimicrobial resistance control approaches [12,19]. The role of MGEs and prevalent across-sectoral transmission highlight the inefficiency of fragmented, single-sector interferences [128,129,130]. Policymakers should be prioritizing integrated surveillance systems that utilize genomic sequencing methods [131,132], improve antimicrobial stewardship, regulate antimicrobial usage in agriculture, and improve sanitation and waste management infrastructure, particularly in resource-limited environments [47,133].

### 3.8. Limitations and Future Directions

The interpretation of findings is constrained by high heterogeneity, potential publication bias, and variability in study design and reporting. Contradictions in analytical units and possible non-independence of observations further limit comparability. Given the predominance of cross-sectional studies, observed associations between MGEs and resistance genes should not be interpreted as evidence of causality or transmission. Future research should focus on standardized methodologies, longitudinal designs, and expanded genomic analyses to better understand the dynamics of resistance across One Health systems.

## 4. Materials and Methods

### 4.1. Study Design and Protocol Registration

The review and meta-analysis followed the Preferred Reporting Items for Systematic Reviews and Meta-Analyses (PRISMA 2020) guidelines [21]. The review protocol was registered with the International Prospective Register of Systematic Reviews (PROSPERO; CRD420251271257) to ensure methodological transparency and reproducibility [134]. The geographical scope followed the United Nations’ macro-regional classification of Africa, which divided Africa into five regions: Eastern, Central, Southern, Northern, and Western [135]. The protocol strictly followed the PICOTS (population, intervention, comparator, outcome, timing and stetting) framework, and this is summarized in Table 13.

### 4.2. Eligibility Criteria

#### 4.2.1. Inclusion Criteria

Studies were considered eligible if they:

1. Reported primary data from African countries;
2. Investigated MGEs and their contribution to AMR within a One Health context;
3. Utilized molecular, genomic, or metagenomic methods to identify MGEs or ARGs;
4. Examined bacterial populations from human, animal, food, or environmental sources;
5. Reported outcomes related to ARG prevalence, MGE–AMR associations, or horizontal gene transfer;
6. Were peer-reviewed articles published in English. No temporal restrictions were applied to maximize evidence capture.

#### 4.2.2. Exclusion Criteria

Studies were excluded if they:

1. Were conducted outside Africa;
2. Focused on non-bacterial organisms;
3. Reported phenotypic resistance without genetic confirmation;
4. Do not explicitly identify MGEs or ARGs;
5. Lacked relevance to One Health transmission pathways;
6. Consisted solely of theoretical models, simulations, editorials, conference abstracts, or review articles.

### 4.3. Search Strategy

A systematic literature search was performed in databases including PubMed, Scopus, and ScienceDirect, for studies published until December 2025. Boolean operators and Medical Subject Headings (MeSH) were applied as follows: (“mobile genetic element” OR plasmid OR transposon OR integron OR bacteriophage OR “horizontal gene transfer”) AND (“antimicrobial resistance” OR “antibiotic resistance” OR ARG) AND (Africa OR “Sub-Saharan Africa” OR names of individual African countries) AND (human* OR animal* OR livestock OR environment OR water OR food). Search strings were adapted for each database to optimize sensitivity. The full database-specific strategies are provided in the Supplementary Materials to support reproducibility. In addition, reference lists of relevant reviews were screened to identify additional eligible studies.

### 4.4. Study Selection

All retrieved records were imported into Rayyan software (SaaS; Cambridge, MA, USA) [136] for screening, and duplicate records were removed. Two independent reviewers conducted title and abstract screening, followed by full-text eligibility assessment and data extraction using Rayyan Software. Inter-reviewer disagreements were resolved through discussion or by a third reviewer when necessary. The study selection process was documented using a PRISMA flow diagram.

### 4.5. Data Extraction

A standardized data extraction form was developed using Rayyan software [136]. Data to be extracted include the author(s)’ name, year of publication, title, DOI, country, African sub-region, study design and setting, sample source (human, animal, or environment), bacterial species, type(s) of mobile genetic elements, resistance genes and antimicrobial classes, laboratory and sequencing methods, and evidence of inter-reservoir transmission. Reference management was performed using Mendeley software (version X7; Thomson Reuters, Toronto, ON, Canada).

### 4.6. Unit of Analysis

The main thing we looked at in this study was the study-level observation, which was a unique dataset that recorded antimicrobial resistance (AMR), mobile genetic elements (MGEs), or related resistance genes (ARGs) in a certain population, setting, or interface. Individual studies may produce multiple observations when results are shared across different bacterial species, sample sources (e.g., human, animal, environment), geographical areas, or laboratory methodologies. These observations were considered separate analytical units to address variability within the study. To reduce the chance of bias caused by non-independence, subgroup analyses and meta-regression were conducted using study-level covariates. The results were then carefully interpreted as descriptive summaries instead of independent effect estimates.

### 4.7. Quality Assessment

Study quality was evaluated using the Cochrane Risk of Bias Tool (RoB 2.0) [137], which was adapted for quality assessment criteria for genomic and metagenomic studies. Three domains were assessed as follows: (1) Selection bias (representativeness of study population); (2) measurement bias (reliability of genomics laboratory testing); (3) reporting bias (completeness and transparency of data). Each study was rated as having low, moderate, or high risk of bias. Discrepancies between reviewers were resolved by consensus.

### 4.8. Data Synthesis and Statistical Analysis

#### 4.8.1. Narrative Synthesis

A narrative synthesis was undertaken to summarize and interpret the characteristics of included studies because substantial methodological and epidemiological heterogeneity was anticipated across study designs, sampling strategies, laboratory methods, and reporting formats. The narrative synthesis aimed to (i) identify mobile genetic elements (MGEs) implicated in antimicrobial resistance (AMR) dissemination, (ii) quantify the distributions of antimicrobial resistance genes (ARGs) and antimicrobial classes linked to MGEs, (iii) identify AMR classes mobilized by MGEs, (iv) describe reported patterns of co-occurrence and potential connectivity of AMR and MGEs across One Health interfaces, based on available observational evidence, and (v) assess regional variation in MGEs and ARGs across Africa.

Data were collected and sorted into preset groups, such as MGE type, ARGs, antimicrobial class, host reservoir (human, animal, or environment), One Health interface, and African sub-region (North, East, West, Central, and Southern Africa). Transmission routes were classified based on established processes, including horizontal gene transfer (HGT), clonal spread, or a combination of these. Cross-interface presence was determined by the reporting of analogous MGEs or ARGs across diverse interfaces, such as human–animal, animal–environment, or human–environment. These observed patterns were interpreted as suggestive of potential connectivity, rather than definitive proof of transmission. Subsequently, cross-tabulation analyses were performed to examine the distribution of ARG families across One Health interfaces and to identify preferred ARG–MGE associations.

The results were then presented in tables and heatmaps. To aid in the interpretation of the narrative, descriptive quantitative summaries were generated, encompassing frequencies, proportions, and mean prevalence estimates. Given the non-normal distribution of the prevalence estimates, a Kruskal–Wallis rank-sum test was employed to assess potential disparities in MGE prevalence across different geographic regions. To reveal regional trends in Africa, geographic distribution maps of ARGs and antimicrobial classes linked to MGEs were generated using the spatial visualizations packages in R software (version 4.5.1).

#### 4.8.2. Meta-Analysis

##### Random-Effects Meta-Analysis

A random-effects meta-analysis of proportions was performed to estimate the pooled prevalence of pathogen occurrence and MGE-associated AMR across studies. Statistical analyses were conducted using R software (version 4.5.1), primarily employing the meta and metafor packages. The DerSimonian–Laird random-effects model was used to account for between-study variability and to generate pooled prevalence estimates with corresponding 95% confidence intervals (CIs). Proportions were transformed where appropriate to stabilize variance prior to pooling. Statistical heterogeneity among studies was assessed using Cochran’s Q test, between-study variance (τ^2^), and the I^2^ statistic. An I^2^ value exceeding 50% was interpreted as substantial heterogeneity; however, given the expected diversity of included studies, higher values were anticipated and interpreted cautiously. Forest plots were produced to visualize individual study estimates and pooled effects.

##### Subgroup Analysis

Subgroup analyses were conducted to explore potential sources of heterogeneity and to compare prevalence estimates across key study characteristics. Subgroups were defined according to bacterial pathogens, antimicrobial classes, geographic regions (North, East, West, Central, and Southern Africa), One Health interfaces (human, animal, environmental, and combined interfaces), and laboratory methods used for MGEs detection. These analyses were performed using random-effects logistic regression models implemented within the metafor package. This allowed estimation of pooled proportions and subgroup-specific effects while accounting for between-study heterogeneity. Statistical differences between subgroups were evaluated using tests for subgroup interaction.

##### Meta-Regression

A mixed-effects meta-regression analysis was performed to identify study-level variables associated with variability in AMR and MGE prevalence. The analysis included data from 91 studies and considered potential moderators such as study design, laboratory method, geographic region, publication year, and the One Health interface. Meta-regression models were fitted using the rma() function in the metafor package, with random effects accounting for unexplained between-study variation. Regression coefficients, 95% confidence intervals, and *p*-values were examined to assess the contribution of each moderator. Residual heterogeneity and explained variance (R^2^) were also evaluated to determine model performance.

##### Publication Bias

Publication bias and small-study effects were assessed using both graphical and statistical approaches. Funnel plots were visually inspected for asymmetry, while Egger’s linear regression test was used to statistically evaluate funnel plot asymmetry. In addition, the trim-and-fill method was applied within a random-effects framework to estimate the potential impact of unpublished or missing studies on pooled estimates. Forest plots and funnel plots were generated using the meta and metafor packages in the R software. The presence of publication bias was considered when interpreting pooled estimates, and results were treated as potentially influenced by selective reporting.

### 4.9. Ethical Considerations and AI Disclosure

Ethical approval was not required, as this study analyzed data derived exclusively from previously published literature. The findings will be disseminated through peer-reviewed publications, scientific conferences, and policy-relevant outputs to support One Health antimicrobial resistance strategies in Africa. The review followed the rules of open science, such as being open and sharing data responsibly. Generative artificial intelligence (GenAI), specifically ChatGPT (OpenAI GPT-5.3), was used solely for language editing, formatting standardization, and alignment with MDPI Antibiotics guidelines; no AI tools were used for data generation, modification, or analysis.

## 5. Conclusions

The studies consolidated existing reports on the role of mobile genetic elements (MGEs) in antimicrobial resistance (AMR) throughout the African continent. It is concluded that plasmids, integrons, and insertion sequence-associated elements are commonly associated with AMR and are probable significant contributors to the spreading of resistance genes. It is further concluded that *bla*CTX-M-15, *sul2*, *bla*TEM, *tet*(*A*), and *qnr* are the commonly detected resistance genes associated with the important antimicrobial classes, such as β-lactams, fluoroquinolones, aminoglycosides, and tetracyclines. However, these findings should be interpreted with caution due to substantial heterogeneity across studies, potential publication bias, and the mainly observational nature of reported data. The simultaneous presence of MGEs and resistance genes indicates the potential for horizontal gene transfer; nonetheless, direct evidence of transmission pathways or causality across One Health interfaces remains limited. Moreover, variations in study design, sampling methods, and reporting practices result in discrepancies in the number of cases observed across different regions and contexts. The results indicated that antimicrobial resistance (AMR) is a significant One Health issue in Africa and underscored critical deficiencies in standardized data and intersectoral collaboration. Enhancing coordinated One Health surveillance via whole-genome sequencing and metagenomic techniques likely improves our comprehension of the dissemination and potential mobility of resistance genes. Future research must prioritize standardized methodologies, improved reporting standards, and comprehensive comparative analyses across human, animal, and environmental interfaces to clarify the dynamics of MGEs associated with AMR.

## Figures and Tables

**Table 1:** Mean prevalence of mobile genetic element (MGE) types Associated with antimicrobial resistance (AMR) studies in Africa.

No	MGE Types	Number of Observations	Mean MGEs Prevalence (%)	Mean AMR Prevalence (%)
1	Plasmid	210	35.4	71.7
2	Integron	89	35.0	54.8
3	Plasmid + Integron	37	42.2	62.9
4	Plasmid + Insertion Sequences	27	95.8	81.0
5	Plasmid + Genomic Islands	13	13.6	59.4
6	Insertion Sequences	12	62.0	100.0
7	Plasmid + Integron + Insertion Sequences + Transposon	8	47.8	93.0
8	Plasmid + Integron + Insertion Sequences + Prophage	7	23.2	7.4

**Table 2:** Mean Prevalence of Antimicrobial Resistance Genes (ARGs) Associated with Mobile Genetic Elements (MGEs).

No	ARGs	Numbers of Observations	Mean ARG Prevalence (%)
1	*sul*2	34	69.6
2	*bla*CTX-M-15	33	78.6
3	*tet*A	30	49.9
4	NR (not reported)	46	52.1
5	*bla*TEM	23	59.1
6	*sul*1	23	69.6
7	*sul3*	22	69.6
8	*dfrA14*	21	25.4
9	*qnrB*	21	18.0

**Table 3:** Mean prevalence of antimicrobial resistance and mobile genetic elements across One Health interfaces.

No	Interface	Numbers of Observations	Mean AMR Prevalence (%)	Mean MGEs Prevalence (%)
1	Human	134	75.1	47.8
2	Animal–Environment	91	54.5	32.8
3	One Health	68	61.3	40.2
4	Environment	70	64.5	22.2
5	Animal	63	58.5	40.7
6	Human–Animal	23	98.9	40.9
7	Human–Environment	12	54.4	86.0

**Table 4:** Evidence of cross-spread and transmission mechanisms across interfaces (frequency).

Interface	Cross-Spread	Transmission Mechanism(s)	Number of Observations
Animal–Environment	Yes	HGT	40
Animal–Environment	Yes	HGT + Clonal	37
Animal	Yes	HGT + Clonal	47
Environment	Yes	HGT	28
Animal–Environment	No	HGT	11
Animal	Yes	HGT	10
Environment	No	HGT	10

**Table 5:** Distribution of antimicrobial resistance gens (ARGs) by One Health Interface.

ARGs	Animal	Animal–Environment	Environment	Human	Human–Animal	Human–Environment	One Health	Total
Other	88	115	74	157	1	0	0	435
*sul*	3	45	7	25	0	0	0	80
*tet*	11	31	9	22	0	0	0	73
*bla*TEM	2	18	6	31	0	0	0	57
*qnr*	6	26	4	28	0	0	0	64
*bla*CTX-M	22	5	20	36	1	0	1	85
*aad*	9	23	6	8	0	0	0	46
*aac*	4	8	3	11	0	0	0	26
*bla*SHV	3	3	3	9	0	0	0	18
*erm*	8	0	0	0	0	0	0	8
*mph*	1	0	0	4	0	0	0	5

**Table 6:** MGE detection rates by laboratory and sequencing method.

No	Lab Method	Number of Observations	MGE Detection Rate (%)
1	Whole-genome sequencing (WGS)	160	100
2	Genomic & Bioinformatic Methods	95	98.9
3	Molecular Detection & Typing	116	87.9
4	Phenotypic Characterization	121	86.8

**Table 7:** Comparison of MGE prevalence by method type.

Method Type	Number of Observations	Mean MGE Prevalence (%)
Conventional/PCR-based	333	36.2
Genomic/Metagenomic	160	38.8

**Table 8:** Mean Prevalence of Mobile Genetic Elements and Antimicrobial Resistance by African Region.

Region	Number of Observations	Mean MGEs Prevalence (%)	Mean AMR Prevalence (%)
North Africa	140	44.1	67.9
East Africa	68	35.2	57.8
West Africa	141	32.6	77.4
Southern Africa	84	32.4	57.7
Central Africa	28	NA	54.7

**Table 9:** Subgroup meta-analysis by bacterial pathogen (random-effects model).

Pathogen Type	Number of Studies	Pooled Proportion (%)	95% CI	τ^2^	Q	I^2^ (%)
*Salmonella enterica*	22	84.53	0.4527–0.9731	17.43	1231.12	98.3
*Escherichia coli*	57	93.24	0.8092–0.9782	16.74	1856.91	97.0
Overall	79	91.49	0.7969–0.9671	17.19	3373.92	97.7

**Table 10:** Subgroup meta-analysis by antimicrobial class (random-effects model).

Antimicrobial Class	Number of Studies	Pooled Proportion (%)	95% CI	τ^2^	Q	I^2^ (%)
Beta-lactams	69	92.68	0.8018–0.9754	19.19	2261.84	97.0
Fluoroquinolones	10	97.48	0.4388–0.9995	26.91	701.67	98.7
Aminoglycosides	7	93.45	0.5733–0.9934	6.96	281.37	97.9
Sulfonamides	2	98.90	0.0471–1.0000	19.90	0.00	0.0
Tetracyclines	3	99.61	0.0009–1.0000	30.99	0.00	0.0
Overall	91	94.20	0.8546–0.9782	19.29	3494.56	97.4

**Table 11:** Subgroup Meta-analysis of MGE-Associated AMR by Geographic Region in Africa.

Region	Number of Studies	Pooled Proportion (%)	95% CI	τ^2^	I^2^ (%)
East Africa	20	74.6	0.407–0.926	9.81	97.3
West Africa	29	99.6	0.877–1.000	35.73	95.9
North Africa	23	95.3	0.724–0.994	19.77	96.1
Southern Africa	14	86.8	0.414–0.984	15.83	98.9
Central Africa	5	60.3	0.220–0.891	3.26	96.6

**Table 12:** Predictors from the Meta-Regression Analysis.

Predictor	Estimate	95% CI	*p*-Value
Cross-sectional epidemiological study	7.009	0.276–13.743	0.042 *
Phenotypic characterization (lab method)	−2.345	−4.362–−0.329	0.024 *
Cross-sectional One Health WGS	−5.938	−11.963–0.088	0.053
Retrospective observational study	4.904	−0.185–9.992	0.059
Year	−0.063	−0.241–0.116	0.484

* Significant at *p* < 0.05.

**Table 13:** PICOTS framework.

PICOTS Component	Description
Population (P)	Bacterial populations originating from humans, animals, food, and environmental sources in African countries, including clinical, community, agricultural, and environmental settings.
Intervention/Exposure (I)	Presence, identification, or characterization of mobile genetic elements (MGEs), including plasmids, integrons, transposons, insertion sequences, integrative and conjugative elements, and bacteriophages associated with antimicrobial resistance genes (ARGs).
Comparator (C)	Not applicable. This review synthesized prevalence, associations, and transmission patterns without comparing intervention versus non-intervention groups.
Outcomes (O)	Identification and frequency of antimicrobial resistance genes (ARGs); prevalence of MGE-associated antimicrobial resistance; evidence of horizontal gene transfer; associations between MGEs and resistant phenotypes or high-risk clones; and evidence of cross-sectoral transmission across human, animal, and environmental interfaces.
Timeframe (T)	No restriction on publication year; all eligible studies available up to the final search date were considered.
Study Design (S)	Observational studies (cross-sectional, cohort, and surveillance); molecular epidemiology studies; whole-genome sequencing (WGS) and metagenomic investigations; and experimental studies providing empirical evidence of MGE-mediated antimicrobial resistance. Systematic and narrative reviews were excluded from quantitative synthesis.

## Data Availability

All data supporting the findings of this study are available within the article and its Supplementary Materials. Extracted data and analytical code (R scripts) are available from the corresponding author upon the reviewer’s and editor’s request.

## Supplementary Materials

The following supporting information can be downloaded at: https://www.mdpi.com/article/10.3390/antibiotics15050456/s1. Supplementary material S1. PRISMA checklist showing study selection process; Figure S1. Risk of bias assessment full result. Figure S2. presents the antimicrobial resistance (AMR) burden across One Health interfaces (human, animal, and environmental), while Figure S3 illustrates the regional distribution of mobile genetic element (MGE) types across Africa. Figure S4 summarizes meta-analysis and trend plots of pathogen prevalence, AMR prevalence, MGE dynamics, and publication bias, including: (a) a forest plot showing pooled estimates of pathogen prevalence across included studies; (b) a forest plot of subgroup analysis for pathogen prevalence; (c) a forest plot illustrating the overall pooled prevalence of AMR; (d) a forest plot of subgroup analysis for AMR prevalence; (e) a forest plot showing regional subgroup prevalence estimates; (f) a forest plot comparing prevalence across One Health interfaces (meta-interface comparisons); (g) a funnel plot assessing potential publication bias among included studies; (h) temporal trend analysis of MGE prevalence over time; (i) temporal trend of antibiotic resistance gene (ARG) prevalence; (j) trends in AMR prevalence across One Health interfaces over time; and (k) a quality-weighted temporal trend analysis of AMR prevalence accounting for study quality and heterogeneity.PROSPERO Registered Protocol [134].

## Abbreviations

AMR	Antimicrobial resistance
ARG	Antimicrobial Resistance Gene
AMRSNET	Africa antibacterial resistance Surveillance Network
AST	Antimicrobial Susceptibility Testing
Β	Regression Coefficient
CLSI	Clinical and Laboratory Standards Institute
CI	Confidence Interval
EUCAST	European Committee on Antimicrobial Susceptibility Testing
ESBL	Extended-Spectrum β-Lactamase
FAO	Food and Agriculture Organization of the United Nations
GI	Genomic Island
GLASS	Global Antibacterial Resistance Surveillance System
HGT	Horizontal Gene Transfer
ICE	Integrative and Conjugative Element
I^2^	I-squared (Measure of Statistical Heterogeneity)
IS	Insertion Sequence
LMICs	Low- and Middle-Income Countries
MDR	Multidrug Resistance
MGE	Mobile Genetic Element
NGS	Next-Generation Sequencing
One Health	Integrated Approach Linking Human, Animal, and Environmental Health
PICOTS	Population, Intervention, Comparator, Outcome, Timeframe, and Setting
PRISMA	Preferred Reporting Items for Systematic Reviews and Meta-Analyses
PROSPERO	International Prospective Register of Systematic Reviews
Q	Cochran’s Heterogeneity Statistic
τ^2^	Between-Study Variance (Tau-squared)
WHO	World Health Organization
WGS	Whole-Genome Sequencing
WOAH	World Organization for Animal Health (formerly OIE)
