# Supplementary material for: Mobile Genetic Elements Associated with Antimicrobial Resistance Across One Health Interfaces in Africa: A Systematic Review and Meta-Analysis
Source: Antibiotics (Basel). 2026 Apr 30;15(5):456. doi: 10.3390/antibiotics15050456 (PMC13203366; doi:10.3390/antibiotics15050456)
Supplement: Supplementary file 1 [file antibiotics-15-00456-s001.zip › PROSPERO Published Protocol.pdf]

NIHR | National Institute for Health and Care Research

PROSPERO
International prospective register of systematic reviews

# The Role of Mobile Genetic Elements in the Spread of Antimicrobial Resistance Across Human, Animal, and Environmental Interfaces in Africa: A Systematic Review

*Kedir A. Hassen, Jose Fafetine, Laurinda Augusto, Inacio Mandomando, Marcelino Garrine, Sileshi Guddina*

**Citation**

Kedir A. Hassen, Jose Fafetine, Laurinda Augusto, Inacio Mandomando, Marcelino Garrine, Sileshi Guddina. The Role of Mobile Genetic Elements in the Spread of Antimicrobial Resistance Across Human, Animal, and Environmental Interfaces in Africa: A Systematic Review. PROSPERO 2026 CRD420251271257. Available from https://www.crd.york.ac.uk/PROSPERO/view/CRD420251271257.

## REVIEW TITLE AND BASIC DETAILS

### Review title

The Role of Mobile Genetic Elements in the Spread of Antimicrobial Resistance Across Human, Animal, and Environmental Interfaces in Africa: A Systematic Review

### Original language title

English

### Review objectives

What is the role of mobile genetic elements in disseminating antimicrobial resistance across human, animal, and environmental reservoirs in Africa?

*Context and rationale*

Antimicrobial resistance (AMR) poses a growing and disproportionate threat to public health, food security, and economic development in Africa. The continent faces unique challenges, including high infectious disease burdens, widespread antimicrobial misuse in human and veterinary medicine, limited regulatory enforcement, and inadequate wastewater and environmental management systems. These conditions create favorable ecological niches for the emergence, maintenance, and dissemination of resistant bacteria.

A key molecular driver of AMR dissemination is horizontal gene transfer (HGT) mediated by mobile genetic elements (MGEs), including plasmids, transposons, integrons, integrative and conjugative elements, and bacteriophages. MGEs enable rapid exchange of antimicrobial resistance genes (ARGs) within and between bacterial populations, facilitating the spread of multidrug-resistant (MDR) and critically resistant pathogens across human, animal, and environmental reservoirs.

In Africa, increasing use of whole-genome sequencing and metagenomic approaches has revealed a complex and interconnected AMR landscape, where MGEs act as molecular bridges linking hospitals, farms, food systems, wastewater, and natural ecosystems. However, evidence remains fragmented across countries, sectors, and bacterial species, with limited synthesis focusing specifically on the African One Health context.

This systematic review aims to consolidate and critically appraise existing evidence on MGE-mediated AMR dissemination in Africa, providing a comprehensive understanding of transmission pathways, resistance platforms, and methodological approaches relevant to surveillance and control.

### Keywords
Antimicrobial resistance; Mobile genetic element; Horizontal Gene Transfer; One Health; Genomics; Africa

## SEARCHING AND SCREENING

### Searches

Electronic databases: PubMed/MEDLINE, Scopus, Web of Science, Embase, CAB Abstracts, ScienceDirect, and SpringerLink. Grey literature will include WHO, FAO, WOAH, Africa CDC reports, and accessible theses or institutional repositories.

Search terms will combine controlled vocabulary and free text related to mobile genetic elements (plasmids, integrons, transposons, bacteriophages, horizontal gene transfer), antimicrobial resistance, Africa, and One Health reservoirs. Search strategies will be adapted for each database.

### Study design

Only nonrandomized study types will be included.

*Included*

- Observational studies (cross-sectional, cohort, surveillance studies)
- Molecular epidemiology studies
- Whole-genome sequencing and metagenomic studies
- Experimental studies reporting MGE-mediated AMR
- English published

*Excluded*

Non English published

Systematic and narrative reviews will be screened for references but excluded from synthesis

### Link to search strategy

A full search strategy is available in the full protocol as described in the *Availability of full protocol* section

## ELIGIBILITY CRITERIA

### Human disease modelled

Studies reporting bacterial isolates or metagenomic samples originating from African countries, including:

• Humans (clinical and community settings)

• Animals (livestock, poultry, aquaculture, companion animals, wildlife)

• Environmental sources (water, wastewater, soil, food, abattoirs)

### Animals/Population

*Included*
Studies reporting bacterial isolates or metagenomic samples originating from African countries, including:

• Humans (clinical and community settings)

• Animals (livestock, poultry, aquaculture, companion animals, wildlife)

• Environmental sources (water, wastewater, soil, food, abattoirs)

*Excluded*
Studies not reported from Africa

### Intervention(s) or exposure(s)

*Included*
Inclusion: Studies identifying mobile genetic elements associated with antimicrobial resistance using molecular, genomic, or metagenomic methods in human, animal, food, or environmental samples from Africa.

*Excluded*
Exclusion: Studies without mobile genetic element assessment, reporting phenotypic resistance only, focusing on non-bacterial organisms, lacking One Health relevance, purely methodological, or conducted outside Africa.

### Comparator(s) or control(s)

This review does not have any comparators

### Other selection criteria or limitations applied

Inclusion limited to English-language studies from Africa reporting MGE-associated antimicrobial resistance using molecular or genomic methods. Phenotypic-only studies, non-bacterial organisms, non–One Health contexts, non-African studies, and secondary literature are excluded.

## OUTCOMES TO BE ANALYSED

### Outcome measure(s)

*Included*
Primary outcomes include detection and characterization of antimicrobial resistance genes associated with mobile genetic elements and evidence of horizontal gene transfer. Secondary outcomes include associations between MGEs and resistant phenotypes or high-risk clones and evidence of AMR transmission across human, animal, food, and environmental reservoirs.

*Excluded*
Studies will be excluded if they do not report antimicrobial resistance outcomes linked to mobile genetic elements, report only phenotypic resistance without genetic evidence, focus on non-bacterial organisms, lack One Health relevance, are purely methodological or theoretical without empirical data, or are secondary literature.

## DATA COLLECTION PROCESS

### Study selection and data extraction

*Procedure for study selection*
All retrieved records will be imported into reference management software and Rayyan for deduplication. Study selection will be conducted in two sequential phases by two independent reviewers.

Phase 1: Title and abstract screening

Titles and abstracts will be screened independently against predefined eligibility criteria to exclude clearly irrelevant studies.

Phase 2: Full-text screening

Full texts of potentially eligible studies will be independently assessed for inclusion.

Disagreements at any stage will be resolved through discussion and consensus. If consensus cannot be reached, a third reviewer will arbitrate. Reasons for exclusion at the full-text stage will be documented. The overall selection process will be summarized using a PRISMA flow diagram.

*Prioritise the exclusion criteria*
To ensure consistency and avoid discrepancies, exclusion criteria will be applied in the following priority order, and the highest-ranked applicable criterion will be recorded as the reason for exclusion.

Phase 1: Title and Abstract Screening (Priority Order)

Not conducted in Africa (study setting outside African countries)

Not relevant to antimicrobial resistance (no AMR outcome reported)

No mobile genetic elements assessed

Non-bacterial organisms only (e.g. viruses, fungi, parasites)

Not a primary research study (reviews, editorials, commentaries)

Not relevant to One Health reservoirs (human, animal, food, or environment)

Phase 2: Full-Text Screening (Priority Order)

Not conducted in Africa

No MGE-associated antimicrobial resistance outcomes

Phenotypic resistance only without molecular or genomic evidence

Purely methodological or theoretical study without empirical data

Non-bacterial organisms only

Insufficient data to extract required outcomes

Non-English publication

Application Across Screening Phases

Where multiple exclusion criteria apply, only the highest-ranked criterion will be recorded as the reason for exclusion. This prioritization will be applied consistently across both screening phases.

*Methods for data extraction*

Data extraction will be performed independently by two reviewers using a standardized, piloted extraction form. Data will be extracted from text, tables, and figures/graphs where applicable. When information is unclear or missing, corresponding authors will be contacted by email to request additional data. Discrepancies between reviewers will be resolved through discussion and

consensus or by consultation with a third reviewer. Extracted data will be cross-checked for accuracy before synthesis.

*Data to be extracted: study design*
Study design (cross-sectional, cohort, surveillance, experimental)

Study setting (clinical, community, farm, abattoir, food chain, environmental)

Sampling strategy and sample size

Laboratory approach (culture-based, PCR, WGS, metagenomics)

Study duration and geographic coverage

*Data to be extracted: animal model*
Animal species (e.g. cattle, poultry, aquaculture species, wildlife, companion animals)

Production system (intensive, semi-intensive, extensive, wild)

Sample type (faeces, tissue, milk, carcass, environmental samples from animal settings)

Where reported: age category and sex of animals

For human and environmental studies, this field will be recorded as "not applicable."

*Data to be extracted: intervention of interest*
Type of mobile genetic element (plasmid, transposon, integron, integrative and conjugative element, bacteriophage)

Associated antimicrobial resistance genes

Bacterial species and strain or clone

Detection method (PCR, sequencing, bioinformatics inference)

Evidence of horizontal gene transfer

*Data to be extracted: primary outcome(s)*
Primary outcomes:

Presence and characterization of antimicrobial resistance genes associated with mobile genetic elements

Evidence of horizontal gene transfer involving MGEs

Data format and type:

Binary/dichotomous data (presence/absence of MGEs and resistance genes)

Categorical data (type of MGE, resistance gene class, bacterial species)

Frequency or proportion data where reported

No transformation of outcome data is planned beyond descriptive summarization.

*Data to be extracted: secondary outcome(s)*
Antimicrobial classes associated with MGE-borne resistance

Reservoir source (human, animal, food, environment)

Evidence of inter-reservoir transmission

Use of genomic or metagenomic methods

Data type: categorical and descriptive quantitative data.

*Data to be extracted: other*
Author(s), year of publication, and journal

Country and African sub-region

Language of publication

Funding source and reported conflicts of interest

Study limitations as reported by authors

## Risk of bias and/or quality assessment
By use of SYRCLE's risk of bias tool

Other criteria, namely:

Joanna Briggs Institute (JBI) Critical Appraisal Checklists and Risk of Bias analysis tools (RoB VIS tool)

*Method for risk of bias and/or quality assessment*

Risk of bias and methodological quality will be assessed independently by two reviewers using study design–appropriate standardized tools. Cross-sectional and observational studies will be appraised using the Joanna Briggs Institute (JBI) Critical Appraisal Checklists, while genomic and molecular epidemiology components will be evaluated using adapted quality criteria focusing on sampling, laboratory methods, and data interpretation.

Each reviewer will assess studies independently, and results will be compared. Any discrepancies will be resolved through discussion and consensus; if disagreement persists, a third reviewer will arbitrate. Quality assessment outcomes will be used to inform interpretation of findings but will not be used as exclusion criteria unless studies are deemed critically flawed.

## PLANNED DATA SYNTHESIS

### Strategy for data synthesis

### Planned approach

A narrative synthesis will be the primary approach for all outcomes due to anticipated heterogeneity in study designs, bacterial species, mobile genetic elements (MGEs), antimicrobial resistance genes (ARGs), sampling sources, and laboratory methods. Studies will be synthesized descriptively by geographical region, One Health compartment (human, animal, environment), bacterial taxa, MGE type, and methodological approach (WGS, metagenomics, PCR-based methods).

A quantitative synthesis (meta-analysis) will be considered only if a sufficient number of studies (≥3) report comparable outcome measures, use similar definitions, and provide extractable data. The decision to perform meta-analysis will be based on clinical, methodological, and statistical homogeneity.

### Effect measure

If meta-analysis is feasible, effect measures will include:

Pooled prevalence estimates (with 95% confidence intervals) for MGE-associated ARGs

Odds ratios (ORs) or risk ratios (RRs) where comparative data between reservoirs or settings are available

### Effect models

A random-effects model will be used for all meta-analyses to account for between-study variability arising from differences in populations, settings, bacterial species, and laboratory methods.

### Heterogeneity

Statistical heterogeneity will be assessed using:

Cochran's Q test

$I^2$ statistic, with values of 25%, 50%, and 75% indicating low, moderate, and high heterogeneity, respectively

Sources of heterogeneity will be explored through subgroup and sensitivity analyses where data permit.

### Other

Subgroup analyses may be conducted by African sub-region, One Health compartment, MGE type, bacterial species, and genomic method used. Sensitivity analyses will be performed by excluding studies at high risk of bias. Where multiple outcomes are assessed, appropriate adjustments for multiple comparisons will be applied. Meta-analysis will not be conducted where heterogeneity is extreme or data are insufficient.

## Analysis of subgroups or subsets

*Subgroup analyses*

Where sufficient data are available, subgroup analyses will be conducted according to:

One Health compartment (human, animal, environmental sources)

African sub-region (North, West, Central, East, Southern Africa)

Type of mobile genetic element (plasmids, integrons, transposons, integrative and conjugative elements, bacteriophages)

Bacterial taxa (e.g. Enterobacterales, Campylobacter, Salmonella, Staphylococcus)

Methodological approach (whole-genome sequencing, metagenomics, PCR-based methods)

Subgroup analyses will be performed only when a sufficient number of comparable studies are available.

*Sensitivity*

Sensitivity analyses will be performed by:

Excluding studies assessed as having high risk of bias or low methodological quality

Excluding studies with small sample sizes or incomplete reporting

Comparing pooled estimates with and without studies using non-genomic detection methods

These analyses will assess the robustness of findings and the influence of study quality and methodology on overall results.

*Publication bias*
Assessment of publication bias will be conducted only if meta-analysis includes at least 10 studies. Where applicable, publication bias will be evaluated using funnel plot symmetry and Egger's regression test. If quantitative assessment is not feasible, potential publication bias will be discussed narrative.

## REVIEW AFFILIATION, FUNDING AND PEER REVIEW

### Review team members
**Mr Kedir A. Hassen** (review guarantor and contact) Eduardo Mondlane University. Mozambique.

No conflict of interest declared.

**Professor Jose Fafetine**. Eduardo Mondlane University. Mozambique.

No conflict of interest declared.

**Professor Laurinda Augusto**. Eduardo Mondlane University. Mozambique.

No conflict of interest declared.

**Dr Inacio Mandomando**. CISM. Mozambique.

No conflict of interest declared.

**Dr Marcelino Garrine**. CISM. Mozambique.

No conflict of interest declared.

**Professor Sileshi Guddina**. Addis Ababa University. Ethiopia.

No conflict of interest declared.

### Named contact
**Mr Kedir A. Hassen** (kedirabdi4@gmail.com). Eduardo Mondlane University. Mozambique.

### Review affiliation
Eduardo Mondlane University

### Funding source
Review has no funding and no agreed support from an academic institution and is done in authors' own time.

## TIMELINE OF THE REVIEW

### Review timeline
Start date: 23 December 2025. End date: 31 March 2026.

### Date of first submission to PROSPERO
23 December 2025

### Date of registration in PROSPERO
12 January 2026

## CURRENT REVIEW STAGE

### Publication of review results
Results of the review will be published in English.

### Stage of the review at this submission

| Review stage | Started | Completed |
|---|---|---|
| Pilot work | ✓ | |
| Formal searching/study identification | ✓ | |
| Screening search results against inclusion criteria | | |
| Data extraction or receipt of IPD | | |
| Risk of bias/quality assessment | | |
| Data synthesis | | |

### Review status
The review is currently planned or ongoing.

## ADDITIONAL INFORMATION

### Review type
Pre-clinical animal intervention review

### PROSPERO version history

- Version 1.0, published 12 Jan 2026

### Review conflict of interest
Declared individual interests are recorded under team member details.. No additional interests are recorded for this review.

### Country
Mozambique; Ethiopia

### Other registration details
Not available

### Medical Subject Headings
Abattoirs; Africa; Animals; Animals, Wild; Anti-Bacterial Agents; Aquaculture; Classification; Drug Resistance, Bacterial; Genomics; Humans; Interspersed Repetitive Sequences; Livestock; Pets; Poultry; Soil; Wastewater; Water

**Disclaimer**

The content of this record displays the information provided by the review team. PROSPERO does not peer review registration records or endorse their content.

PROSPERO accepts and posts the information provided in good faith; responsibility for record content rests with the review team. The guarantor for this record has affirmed that the information provided is truthful and that they understand that deliberate provision of inaccurate information may be construed as scientific misconduct.

PROSPERO does not accept any liability for the content provided in this record or for its use. Readers use the information provided in this record at their own risk.

Any enquiries about the record should be referred to the named review contact
