# Supplementary material for: Mobile Genetic Elements Associated with Antimicrobial Resistance Across One Health Interfaces in Africa: A Systematic Review and Meta-Analysis
Source: Antibiotics (Basel). 2026 Apr 30;15(5):456. doi: 10.3390/antibiotics15050456 (PMC13203366; doi:10.3390/antibiotics15050456)
Supplement: Supplementary file 1 [file antibiotics-15-00456-s001.zip › Supplementary Figure_Updated.pdf]

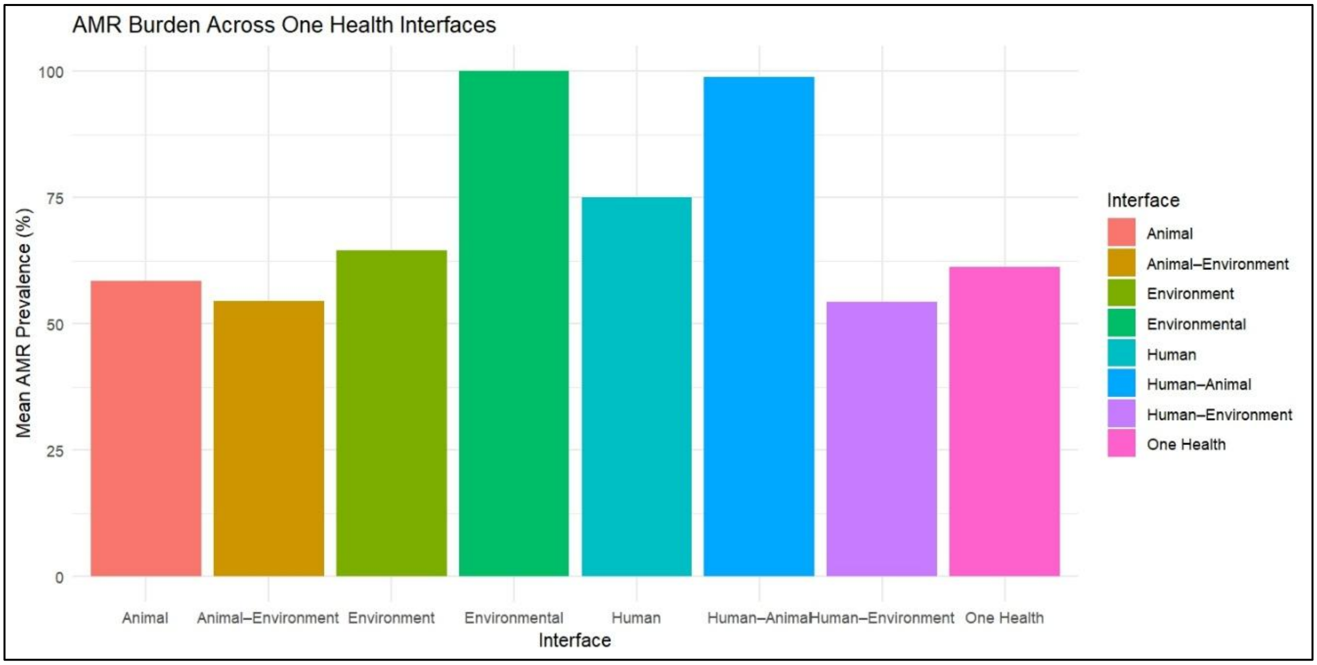

Supplementary Figure S2. AMR Burden Across One Health Interfaces

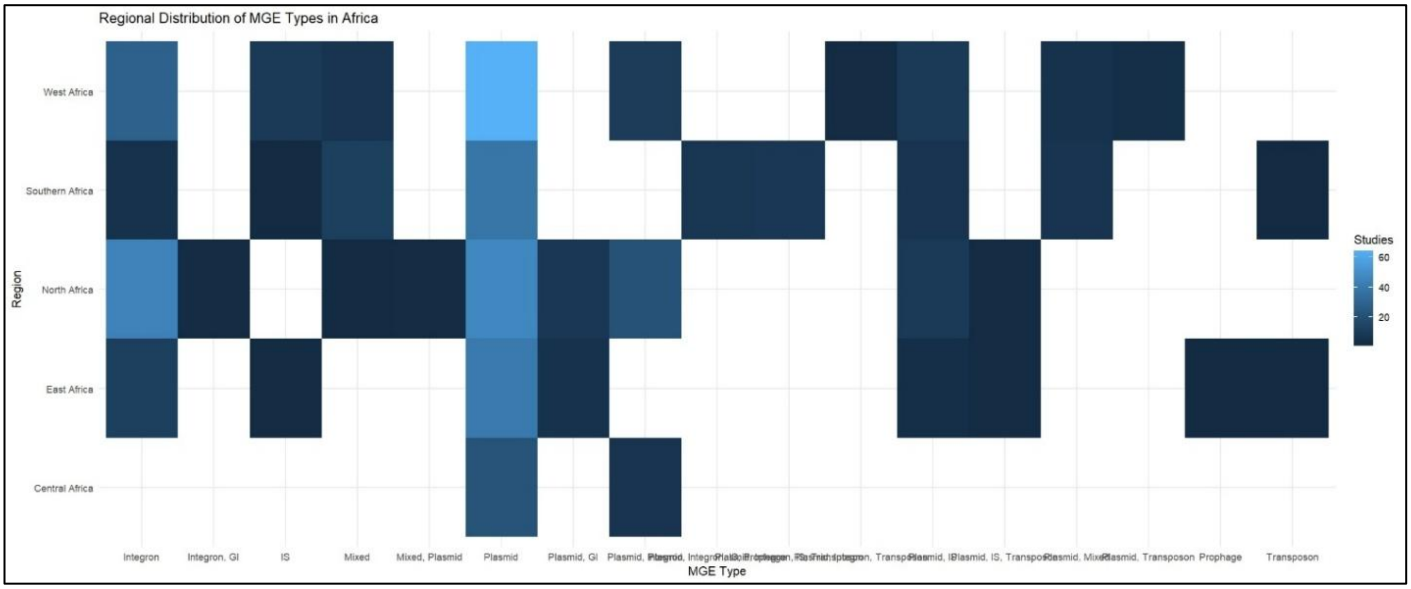

Supplementary Figure S3. Regional Distribution of MGE Types in Africa

Study	Positive	Total	Proportion	95%-CI	Weight
Cuypers et al. 2023	284	284	1.00	[0.99; 1.00]	0.7%
Abaasa N. et al. 2024	47	47	1.00	[0.92; 1.00]	0.7%
Milenkov et al. 2024	86	289	0.30	[0.25; 0.35]	1.4%
Milenkov et al. 2024	140	246	0.57	[0.50; 0.63]	1.4%
Milenkov et al. 2024	28	28	1.00	[0.88; 1.00]	0.7%
Odoch T. et al. 2018	85	133	0.64	[0.55; 0.72]	1.4%
Aworh MK et al. 2021	110	429	0.26	[0.22; 0.30]	1.4%
Yahiaoui M. et al. 2015	70	150	0.47	[0.38; 0.55]	1.4%
Adelowo O.O. et al. 2014	30	36	0.83	[0.67; 0.94]	1.3%
Langata LM et al. 2019	85	150	0.57	[0.48; 0.65]	1.4%
Langata LM et al. 2019	18	150	0.12	[0.07; 0.18]	1.4%
Mashe T et al. 2021	760	784	0.97	[0.95; 0.98]	1.4%
Van Puyvelde S et al. 2019	54	81	0.67	[0.55; 0.77]	1.4%
Naidoo Y, et al. 2024	18	18	1.00	[0.81; 1.00]	0.7%
Muriuki CW et al. 2022	23	95	0.24	[0.16; 0.34]	1.4%
Mohammed R et al. 2024	24	33	0.73	[0.54; 0.87]	1.3%
Odih EE et al. 2022	63	63	1.00	[0.94; 1.00]	0.7%
Adesoji AT, et al., 2016	168	168	1.00	[0.98; 1.00]	0.7%
Touglo K. et al. 2025	7	285	0.02	[0.01; 0.05]	1.3%
Touglo K. et al. 2025	94	285	0.33	[0.28; 0.39]	1.4%
Ed-Dra A et al. 2018	22	26	0.85	[0.65; 0.96]	1.2%
Kariuki K et al. 2023	150	188	0.80	[0.73; 0.85]	1.4%
Abbassi MS et al. 2021	83	83	1.00	[0.96; 1.00]	0.7%
Kikuvi GM, et al. 2010	16	116	0.14	[0.08; 0.21]	1.4%
Rabiu AG et al. 2025	17	25	0.68	[0.46; 0.85]	1.3%
Barguigua A et al. 2019	28	28	1.00	[0.88; 1.00]	0.7%
Kasiano P et al. 2024	16	35	0.46	[0.29; 0.63]	1.3%
Igbinosa IH 2015	80	150	0.53	[0.45; 0.62]	1.4%
Messaili C, et al. 2019	59	100	0.59	[0.49; 0.69]	1.4%
Breurec S et al. 2019	180	582	0.31	[0.27; 0.35]	1.4%
Prah I et al. 2021	62	62	1.00	[0.94; 1.00]	0.7%
Saraiva MdMS et al. 2022	19	19	1.00	[0.82; 1.00]	0.7%
Andrews Sah, et al. 2022	254	254	1.00	[0.99; 1.00]	0.7%
Negeri AA et al. 2023	100	200	0.50	[0.43; 0.57]	1.4%
Akinyemi KO et al. 2023	48	2522	0.02	[0.01; 0.03]	1.4%
Leinyuy JF, et al. 2023	200	200	1.00	[0.98; 1.00]	0.7%
Ball TA et al. 2019	400	400	1.00	[0.99; 1.00]	0.7%
Inwezerua C et al. 2014	114	114	1.00	[0.97; 1.00]	0.7%
Estaleva CE L et al. 2021	75	230	0.33	[0.27; 0.39]	1.4%
Soufi L et al. 2009	60	100	0.60	[0.50; 0.70]	1.4%
Ajibola A.T. et al. 2025	71	71	1.00	[0.95; 1.00]	0.7%
Khalifa HO et al. 2019	126	126	1.00	[0.97; 1.00]	0.7%
Adesoji AT, et al., 2019	69	181	0.38	[0.31; 0.46]	1.4%
Mbelle NM et al. 2019	175	188	0.93	[0.88; 0.96]	1.3%
Afunwa RA et al. 2011	145	145	1.00	[0.97; 1.00]	0.7%
Sunmonu GT et al. 2025	14	14	1.00	[0.77; 1.00]	0.7%
Sunmonu GT et al. 2025	14	14	1.00	[0.77; 1.00]	0.7%
Adesoji AT et al. 2015	29	105	0.28	[0.19; 0.37]	1.4%
Al-Gallas N et al. 2021	42	42	1.00	[0.92; 1.00]	0.7%
Adenipekun EO et al. 2019	144	144	1.00	[0.97; 1.00]	0.7%
Shawa M et al. 2021	36	36	1.00	[0.90; 1.00]	0.7%
Shawa M et al. 2022	56	56	1.00	[0.94; 1.00]	0.7%
Katakweba A.A.S. et al. 2018	390	485	0.80	[0.77; 0.84]	1.4%

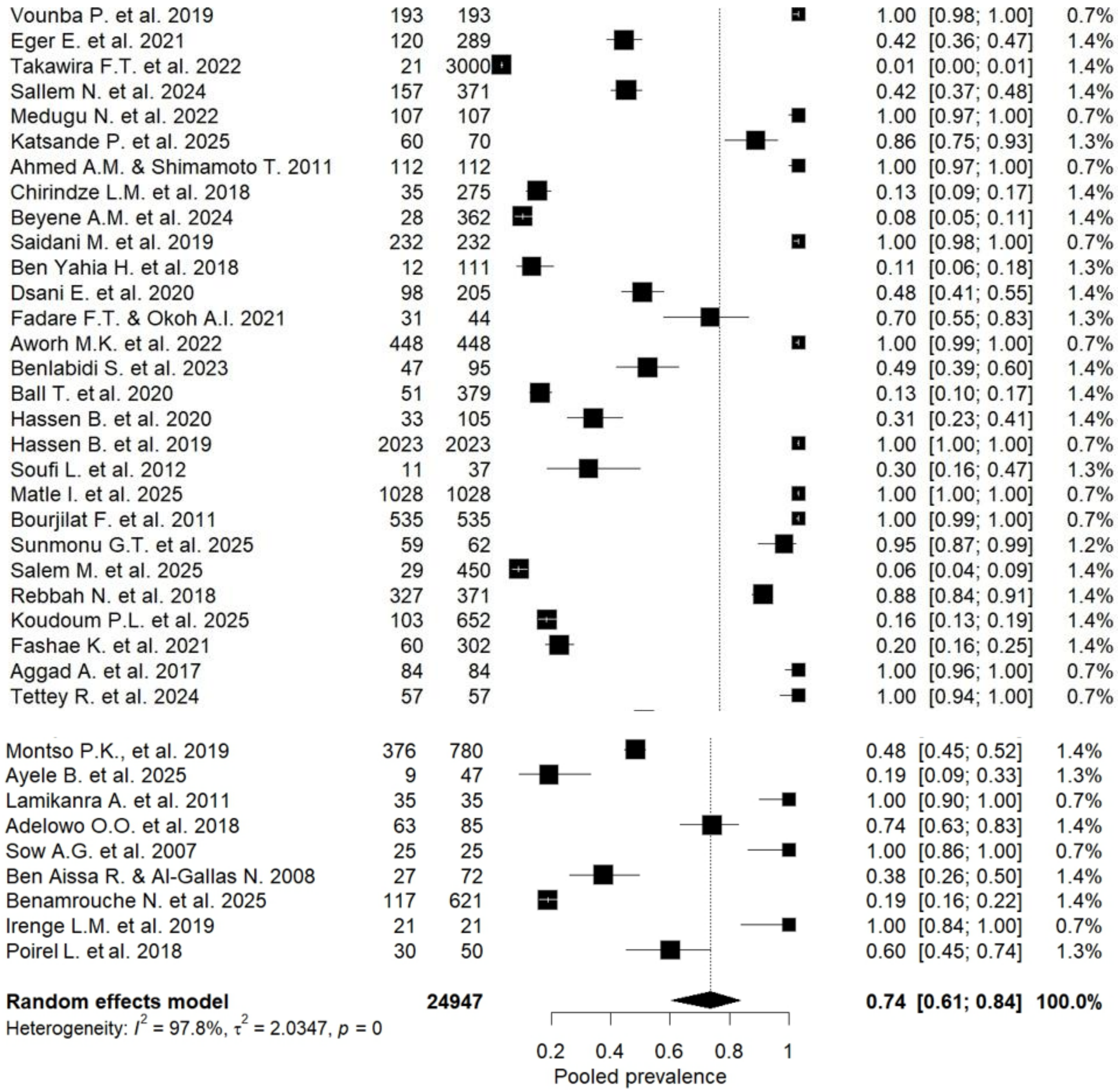

Supplementary Figure S4a. Forest Plot of Pathogen Prevalence

Study	Events	Total	Proportion	95%-CI
**Pathogen_Type = Salmonella enterica**				
Cuypers et al. 2023	284	284	1.00	[0.99; 1.00]
Odoch T. et al. 2018	85	133	0.64	[0.55; 0.72]
Langata LM et al. 2019	18	150	0.12	[0.07; 0.18]
Mashe T et al. 2021	760	784	0.97	[0.95; 0.98]
Odih EE et al. 2022	63	63	1.00	[0.94; 1.00]
Kikuvi GM, et al. 2010	16	116	0.14	[0.08; 0.21]
Kasiano P et al. 2024	16	35	0.46	[0.29; 0.63]
Igbinosa IH 2015	80	150	0.53	[0.45; 0.62]
Akinyemi KO et al. 2023	48	2522	0.02	[0.01; 0.03]
Sunmonu GT et al. 2025	14	14	1.00	[0.77; 1.00]
Adesoji AT et al. 2015	29	105	0.28	[0.19; 0.37]
Al-Gallas N et al. 2021	42	42	1.00	[0.92; 1.00]
Shawa M et al. 2022	56	56	1.00	[0.94; 1.00]
Beyene A.M. et al. 2024	28	362	0.08	[0.05; 0.11]
Ball T. et al. 2020	51	379	0.13	[0.10; 0.17]
Soufi L. et al. 2012	11	37	0.30	[0.16; 0.47]
Matle I. et al. 2025	1028	1028	1.00	[1.00; 1.00]
Sunmonu G.T. et al. 2025	59	62	0.95	[0.87; 0.99]
Salem M. et al. 2025	29	450	0.06	[0.04; 0.09]
Sow A.G. et al. 2007	25	25	1.00	[0.86; 1.00]
Ben Aissa R. & Al-Gallas N. 2008	27	72	0.38	[0.26; 0.50]
Benamrouche N. et al. 2025	117	621	0.19	[0.16; 0.22]
**Random effects model**		**7490**	**0.85**	**[0.45; 0.97]**
Heterogeneity: $I^2 = 98.3\%$, $\tau^2 = 17.4293$, $p < 0.0001$				
**Pathogen_Type = Escherichia coli**				
Abaasa N. et al. 2024	47	47	1.00	[0.92; 1.00]
Milenkov et al. 2024	86	289	0.30	[0.25; 0.35]
Milenkov et al. 2024	140	246	0.57	[0.50; 0.63]
Milenkov et al. 2024	28	28	1.00	[0.88; 1.00]
Aworh MK et al. 2021	110	429	0.26	[0.22; 0.30]
Yahiaoui M. et al. 2015	70	150	0.47	[0.38; 0.55]
Adelowo O.O. et al. 2014	30	36	0.83	[0.67; 0.94]
Langata LM et al. 2019	85	150	0.57	[0.48; 0.65]
Muriuki CW et al. 2022	23	95	0.24	[0.16; 0.34]
Mohammed R et al. 2024	24	33	0.73	[0.54; 0.87]
Touglo K. et al. 2025	94	285	0.33	[0.28; 0.39]
Kariuki K et al. 2023	150	188	0.80	[0.73; 0.85]
Abbassi MS et al. 2021	83	83	1.00	[0.96; 1.00]
Rabiu AG et al. 2025	17	25	0.68	[0.46; 0.85]
Barguigua A et al. 2019	28	28	1.00	[0.88; 1.00]
Messaili C, et al. 2019	59	100	0.59	[0.49; 0.69]
Negeri AA et al. 2023	100	200	0.50	[0.43; 0.57]
Leinyuy JF, et al. 2023	200	200	1.00	[0.98; 1.00]
Ball TA et al. 2019	400	400	1.00	[0.99; 1.00]
Inwezerua C et al. 2014	114	114	1.00	[0.97; 1.00]
Estaleva CE L et al. 2021	75	230	0.33	[0.27; 0.39]
Soufi L et al. 2009	60	100	0.60	[0.50; 0.70]
Ajibola A.T. et al. 2025	71	71	1.00	[0.95; 1.00]
Adesoji AT, et al., 2019	69	181	0.38	[0.31; 0.46]
Mbelle NM et al. 2019	175	188	0.93	[0.88; 0.96]
Afunwa RA et al. 2011	145	145	1.00	[0.97; 1.00]
Sunmonu GT et al. 2025	14	14	1.00	[0.77; 1.00]

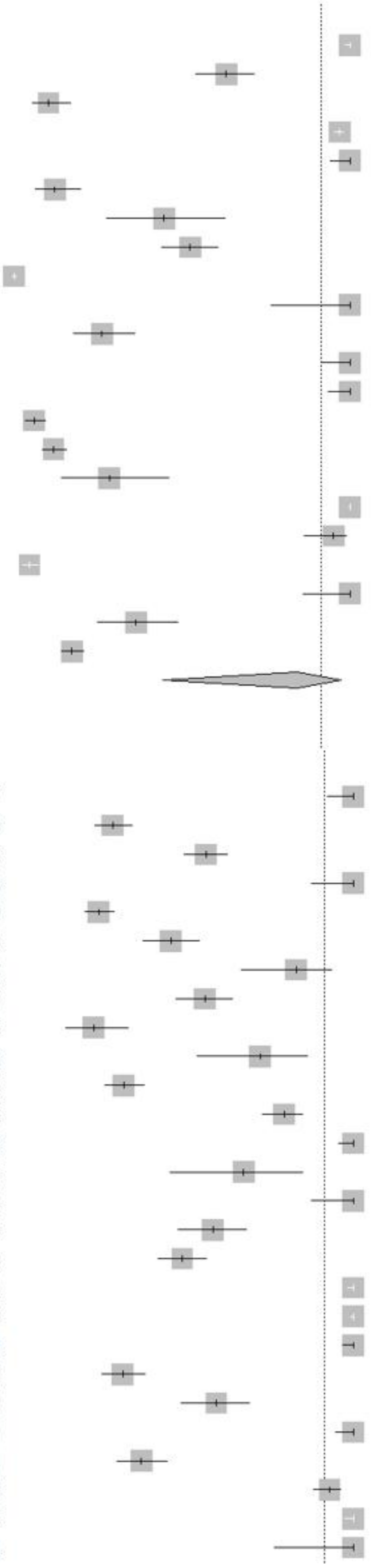

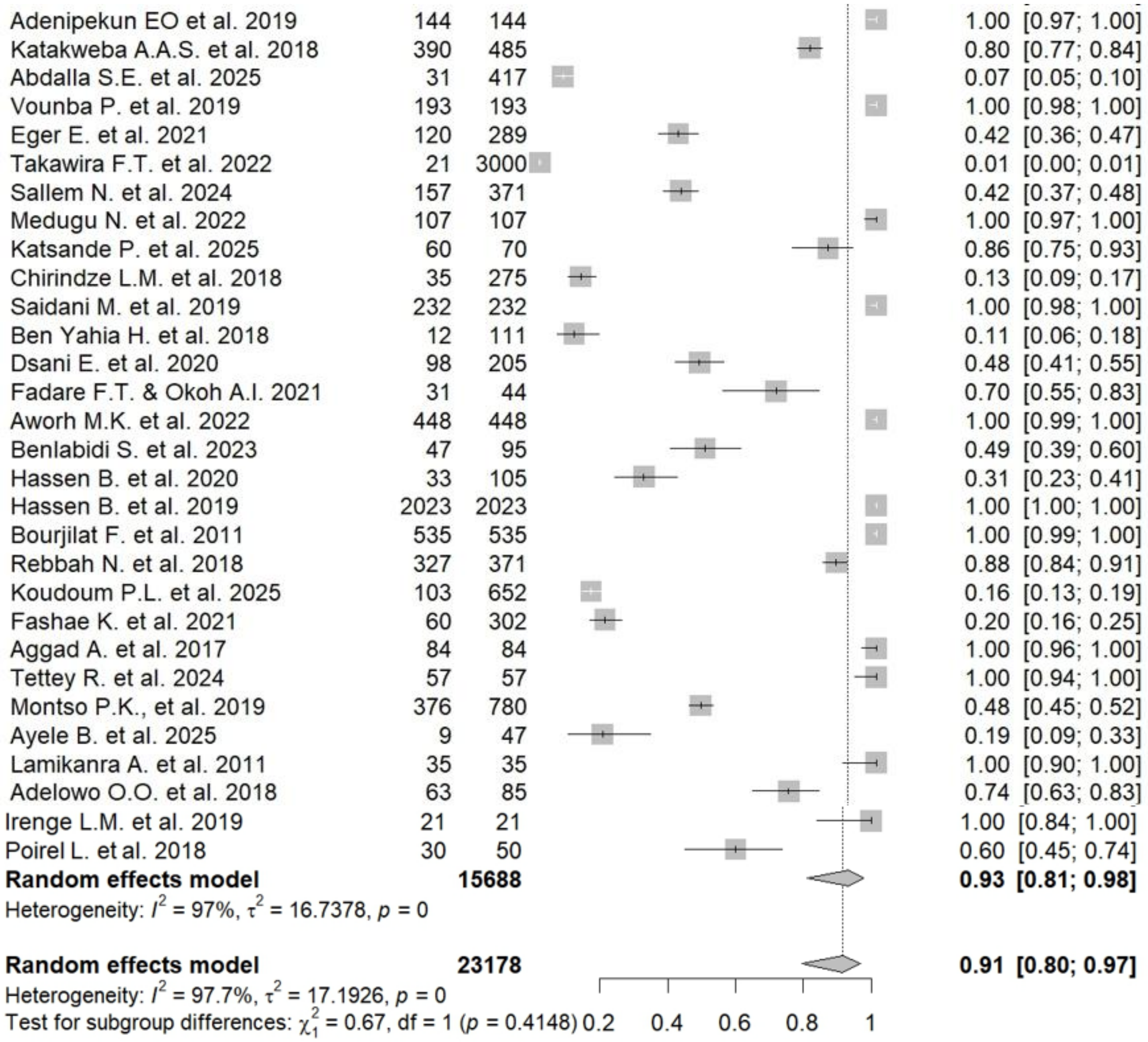

Supplementary Figure S4b. Forest Plot of Subgroup Pathogen Prevalence

Study	Events	Total	Proportion	95%-CI
Cuypers et al. 2023	284	284	1.00	[0.99; 1.00]
Abaasa N. et al. 2024	47	47	1.00	[0.92; 1.00]
Milenkov et al. 2024	86	289	0.30	[0.25; 0.35]
Milenkov et al. 2024	140	246	0.57	[0.50; 0.63]
Milenkov et al. 2024	28	28	1.00	[0.88; 1.00]
Odoch T. et al. 2018	85	133	0.64	[0.55; 0.72]
Aworh MK et al. 2021	110	429	0.26	[0.22; 0.30]
Yahiaoui M. et al. 2015	70	150	0.47	[0.38; 0.55]
Adelowo O.O. et al. 2014	30	36	0.83	[0.67; 0.94]
Langata LM et al. 2019	85	150	0.57	[0.48; 0.65]
Langata LM et al. 2019	18	150	0.12	[0.07; 0.18]
Mashe T et al. 2021	760	784	0.97	[0.95; 0.98]
Van Puyvelde S et al. 2019	54	81	0.67	[0.55; 0.77]
Naidoo Y, et al. 2024	18	18	1.00	[0.81; 1.00]
Muriuki CW et al. 2022	23	95	0.24	[0.16; 0.34]
Mohammed R et al. 2024	24	33	0.73	[0.54; 0.87]
Odih EE et al. 2022	63	63	1.00	[0.94; 1.00]
Adesoji AT, et al., 2016	168	168	1.00	[0.98; 1.00]
Touglo K. et al. 2025	7	285	0.02	[0.01; 0.05]
Touglo K. et al. 2025	94	285	0.33	[0.28; 0.39]
Ed-Dra A et al. 2018	22	26	0.85	[0.65; 0.96]
Kariuki K et al. 2023	150	188	0.80	[0.73; 0.85]
Abbassi MS et al. 2021	83	83	1.00	[0.96; 1.00]
Kikuvi GM, et al. 2010	16	116	0.14	[0.08; 0.21]
Rabiu AG et al. 2025	17	25	0.68	[0.46; 0.85]
Barguigua A et al. 2019	28	28	1.00	[0.88; 1.00]
Kasiano P et al. 2024	16	35	0.46	[0.29; 0.63]
Igbinosa IH 2015	80	150	0.53	[0.45; 0.62]
Messaili C, et al. 2019	59	100	0.59	[0.49; 0.69]
Breurec S et al. 2019	180	582	0.31	[0.27; 0.35]
Prah I et al. 2021	62	62	1.00	[0.94; 1.00]
Saraiva MdMS et al. 2022	19	19	1.00	[0.82; 1.00]
Andrews Sah, et al. 2022	254	254	1.00	[0.99; 1.00]
Negeri AA et al. 2023	100	200	0.50	[0.43; 0.57]
Akinyemi KO et al. 2023	48	2522	0.02	[0.01; 0.03]
Leinyuy JF, et al. 2023	200	200	1.00	[0.98; 1.00]
Ball TA et al. 2019	400	400	1.00	[0.99; 1.00]
Inwezerua C et al. 2014	114	114	1.00	[0.97; 1.00]
Estaleva CE L et al. 2021	75	230	0.33	[0.27; 0.39]
Soufi L et al. 2009	60	100	0.60	[0.50; 0.70]
Ajibola A.T. et al. 2025	71	71	1.00	[0.95; 1.00]
Khalifa HO et al. 2019	126	126	1.00	[0.97; 1.00]
Adesoji AT, et al., 2019	69	181	0.38	[0.31; 0.46]
Mbelle NM et al. 2019	175	188	0.93	[0.88; 0.96]
Afunwa RA et al. 2011	145	145	1.00	[0.97; 1.00]
Sunmonu GT et al. 2025	14	14	1.00	[0.77; 1.00]
Sunmonu GT et al. 2025	14	14	1.00	[0.77; 1.00]
Adesoji AT et al. 2015	29	105	0.28	[0.19; 0.37]
Al-Gallas N et al. 2021	42	42	1.00	[0.92; 1.00]
Adenipekun EO et al. 2019	144	144	1.00	[0.97; 1.00]
Shawa M et al. 2021	36	36	1.00	[0.90; 1.00]
Shawa M et al. 2022	56	56	1.00	[0.94; 1.00]
Katakweba A.A.S. et al. 2018	390	485	0.80	[0.77; 0.84]
Abdalla S.E. et al. 2025	31	417	0.07	[0.05; 0.10]

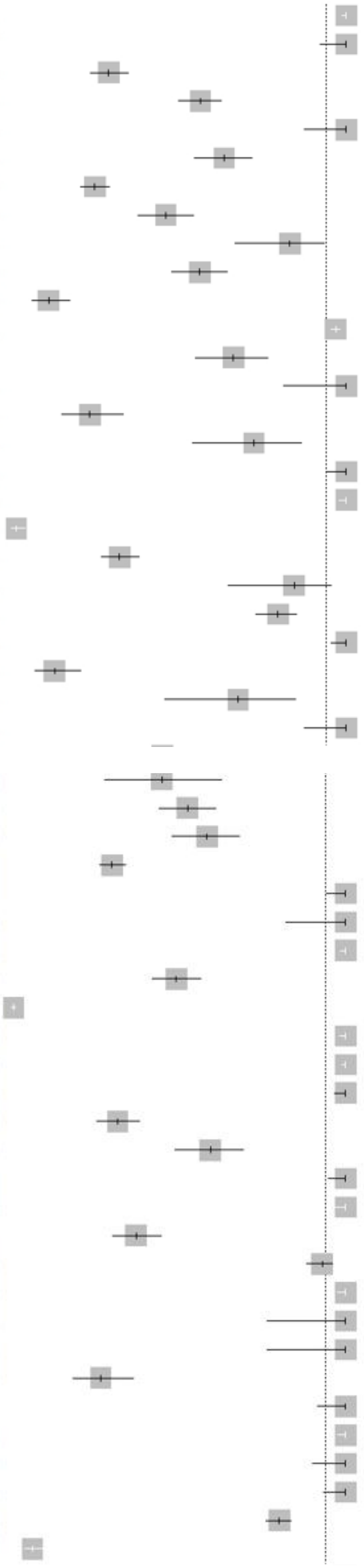

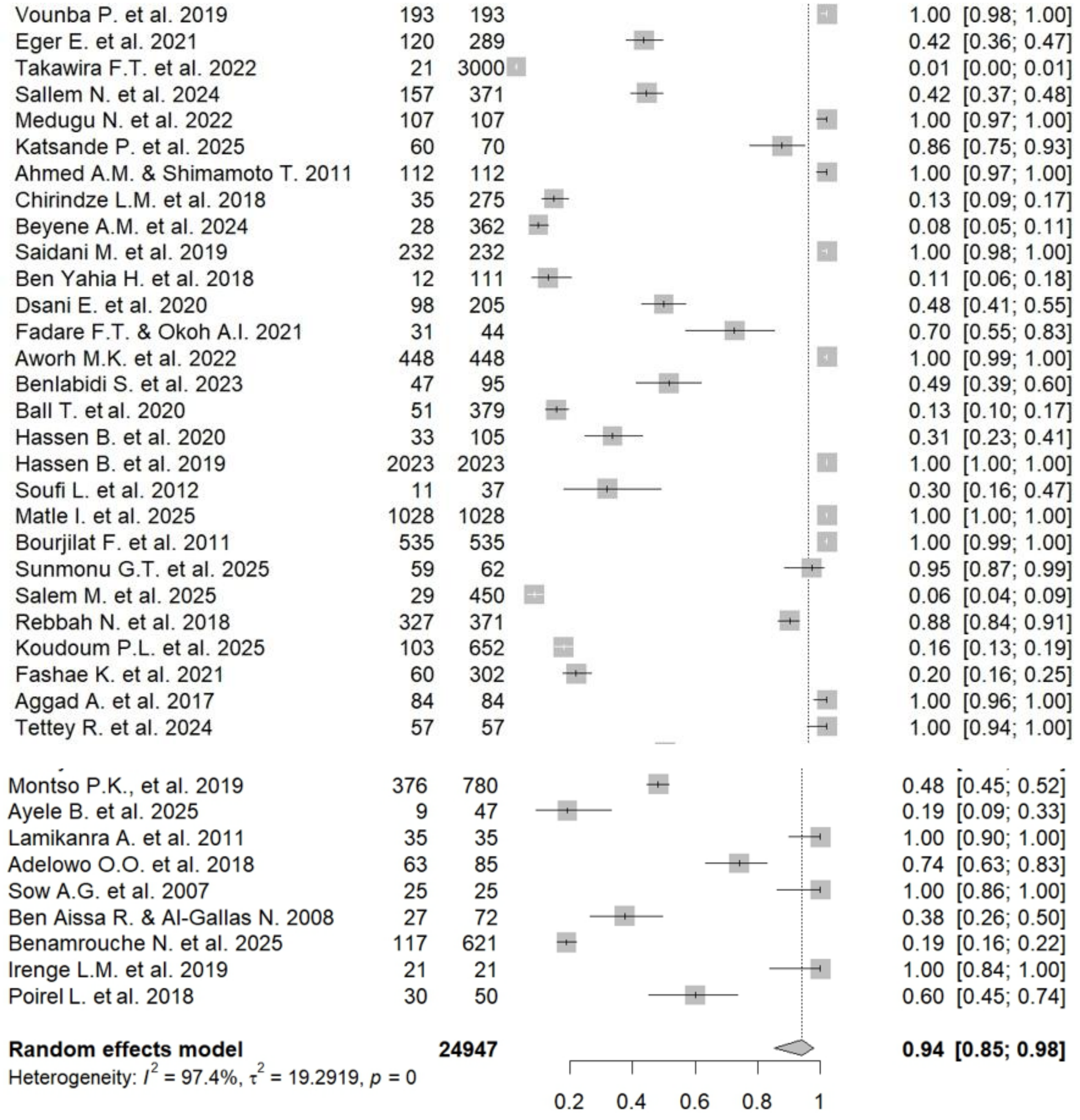

Supplementary Figure S4c. Forest Plot of AMR Prevalence

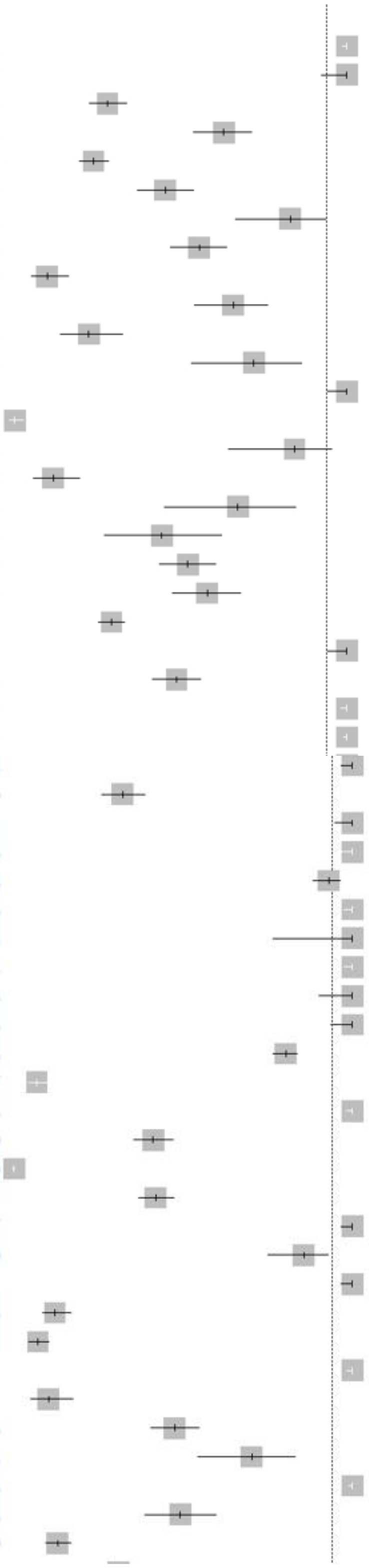

Study	Events	Total	Proportion	95%-CI
**AMR_Class = Beta-lactams**				
Cuypers et al. 2023	284	284	1.00	[0.99; 1.00]
Abaasa N. et al. 2024	47	47	1.00	[0.92; 1.00]
Milenkov et al. 2024	86	289	0.30	[0.25; 0.35]
Odoch T. et al. 2018	85	133	0.64	[0.55; 0.72]
Aworh MK et al. 2021	110	429	0.26	[0.22; 0.30]
Yahiaoui M. et al. 2015	70	150	0.47	[0.38; 0.55]
Adelowo O.O. et al. 2014	30	36	0.83	[0.67; 0.94]
Langata LM et al. 2019	85	150	0.57	[0.48; 0.65]
Langata LM et al. 2019	18	150	0.12	[0.07; 0.18]
Van Puyvelde S et al. 2019	54	81	0.67	[0.55; 0.77]
Muriuki CW et al. 2022	23	95	0.24	[0.16; 0.34]
Mohammed R et al. 2024	24	33	0.73	[0.54; 0.87]
Odih EE et al. 2022	63	63	1.00	[0.94; 1.00]
Touglo K. et al. 2025	7	285	0.02	[0.01; 0.05]
Ed-Dra A et al. 2018	22	26	0.85	[0.65; 0.96]
Kikuvi GM, et al. 2010	16	116	0.14	[0.08; 0.21]
Rabiu AG et al. 2025	17	25	0.68	[0.46; 0.85]
Kasiano P et al. 2024	16	35	0.46	[0.29; 0.63]
Igbinosa IH 2015	80	150	0.53	[0.45; 0.62]
Messaili C, et al. 2019	59	100	0.59	[0.49; 0.69]
Breurec S et al. 2019	180	582	0.31	[0.27; 0.35]
Prah I et al. 2021	62	62	1.00	[0.94; 1.00]
Negeri AA et al. 2023	100	200	0.50	[0.43; 0.57]
Leinyuy JF, et al. 2023	200	200	1.00	[0.98; 1.00]
Ball TA et al. 2019	400	400	1.00	[0.99; 1.00]
Inwezerua C et al. 2014	114	114	1.00	[0.97; 1.00]
Estaleva CE L et al. 2021	75	230	0.33	[0.27; 0.39]
Ajibola A.T. et al. 2025	71	71	1.00	[0.95; 1.00]
Khalifa HO et al. 2019	126	126	1.00	[0.97; 1.00]
Mbelle NM et al. 2019	175	188	0.93	[0.88; 0.96]
Afunwa RA et al. 2011	145	145	1.00	[0.97; 1.00]
Sunmonu GT et al. 2025	14	14	1.00	[0.77; 1.00]
Adenipekun EO et al. 2019	144	144	1.00	[0.97; 1.00]
Shawa M et al. 2021	36	36	1.00	[0.90; 1.00]
Shawa M et al. 2022	56	56	1.00	[0.94; 1.00]
Katakweba A.A.S. et al. 2018	390	485	0.80	[0.77; 0.84]
Abdalla S.E. et al. 2025	31	417	0.07	[0.05; 0.10]
Vounba P. et al. 2019	193	193	1.00	[0.98; 1.00]
Eger E. et al. 2021	120	289	0.42	[0.36; 0.47]
Takawira F.T. et al. 2022	21	3000	0.01	[0.00; 0.01]
Sallem N. et al. 2024	157	371	0.42	[0.37; 0.48]
Medugu N. et al. 2022	107	107	1.00	[0.97; 1.00]
Katsande P. et al. 2025	60	70	0.86	[0.75; 0.93]
Ahmed A.M. & Shimamoto T. 2011	112	112	1.00	[0.97; 1.00]
Chirindze L.M. et al. 2018	35	275	0.13	[0.09; 0.17]
Beyene A.M. et al. 2024	28	362	0.08	[0.05; 0.11]
Saidani M. et al. 2019	232	232	1.00	[0.98; 1.00]
Ben Yahia H. et al. 2018	12	111	0.11	[0.06; 0.18]
Dsani E. et al. 2020	98	205	0.48	[0.41; 0.55]
Fadare F.T. & Okoh A.I. 2021	31	44	0.70	[0.55; 0.83]
Aworh M.K. et al. 2022	448	448	1.00	[0.99; 1.00]
Benlabidi S. et al. 2023	47	95	0.49	[0.39; 0.60]
Ball T. et al. 2020	51	379	0.13	[0.10; 0.17]

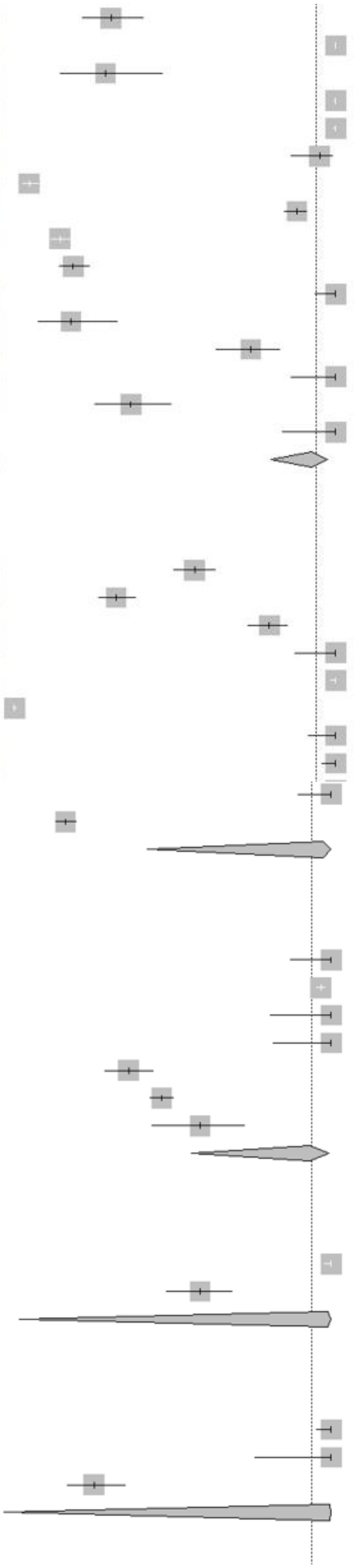

Hassen B. et al. 2020	33	105	0.31 [0.23; 0.41]
Hassen B. et al. 2019	2023	2023	1.00 [1.00; 1.00]
Soufi L. et al. 2012	11	37	0.30 [0.16; 0.47]
Matle I. et al. 2025	1028	1028	1.00 [1.00; 1.00]
Bourjilat F. et al. 2011	535	535	1.00 [0.99; 1.00]
Sunmonu G.T. et al. 2025	59	62	0.95 [0.87; 0.99]
Salem M. et al. 2025	29	450	0.06 [0.04; 0.09]
Rebbah N. et al. 2018	327	371	0.88 [0.84; 0.91]
Koudoum P.L. et al. 2025	103	652	0.16 [0.13; 0.19]
Fashae K. et al. 2021	60	302	0.20 [0.16; 0.25]
Tettey R. et al. 2024	57	57	1.00 [0.94; 1.00]
Ayele B. et al. 2025	9	47	0.19 [0.09; 0.33]
Adelowo O.O. et al. 2018	63	85	0.74 [0.63; 0.83]
Sow A.G. et al. 2007	25	25	1.00 [0.86; 1.00]
Ben Aissa R. & Al-Gallas N. 2008	27	72	0.38 [0.26; 0.50]
Irenge L.M. et al. 2019	21	21	1.00 [0.84; 1.00]
**Random effects model**		**18312**	**0.93 [0.80; 0.98]**
Heterogeneity: $I^2 = 97\%$, $\tau^2 = 19.1891$, $p = 0$			
**AMR_Class = Fluoroquinolones**			
Milenkov et al. 2024	140	246	0.57 [0.50; 0.63]
Touglo K. et al. 2025	94	285	0.33 [0.28; 0.39]
Kariuki K et al. 2023	150	188	0.80 [0.73; 0.85]
Barguigua A et al. 2019	28	28	1.00 [0.88; 1.00]
Andrews Sah, et al. 2022	254	254	1.00 [0.99; 1.00]
Akinyemi KO et al. 2023	48	2522	0.02 [0.01; 0.03]
Al-Gallas N et al. 2021	42	42	1.00 [0.92; 1.00]
Aggad A. et al. 2017	84	84	1.00 [0.96; 1.00]
Lamikanra A. et al. 2011	35	35	1.00 [0.90; 1.00]
Benamrouche N. et al. 2025	117	621	0.19 [0.16; 0.22]
**Random effects model**		**4305**	**0.97 [0.44; 1.00]**
Heterogeneity: $I^2 = 98.7\%$, $\tau^2 = 26.9064$, $p < 0.0001$			
**AMR_Class = Aminoglycosides**			
Milenkov et al. 2024	28	28	1.00 [0.88; 1.00]
Mashe T et al. 2021	760	784	0.97 [0.95; 0.98]
Naidoo Y, et al. 2024	18	18	1.00 [0.81; 1.00]
Saraiva MdMS et al. 2022	19	19	1.00 [0.82; 1.00]
Adesoji AT, et al., 2019	69	181	0.38 [0.31; 0.46]
Montso P.K., et al. 2019	376	780	0.48 [0.45; 0.52]
Poirel L. et al. 2018	30	50	0.60 [0.45; 0.74]
**Random effects model**		**1860**	**0.93 [0.57; 0.99]**
Heterogeneity: $I^2 = 97.9\%$, $\tau^2 = 6.9600$, $p < 0.0001$			
**AMR_Class = Sulfonamides**			
Adesoji AT, et al., 2016	168	168	1.00 [0.98; 1.00]
Soufi L et al. 2009	60	100	0.60 [0.50; 0.70]
**Random effects model**		**268**	**0.99 [0.05; 1.00]**
Heterogeneity: $I^2 = 0\%$, $\tau^2 = 19.9023$, $p = 0.9996$			
**AMR_Class = Tetracyclines**			
Abbassi MS et al. 2021	83	83	1.00 [0.96; 1.00]
Sunmonu GT et al. 2025	14	14	1.00 [0.77; 1.00]
Adesoji AT et al. 2015	29	105	0.28 [0.19; 0.37]
**Random effects model**		**202**	**1.00 [0.00; 1.00]**
Heterogeneity: $I^2 = 0\%$, $\tau^2 = 30.9910$, $p = 1.0000$			

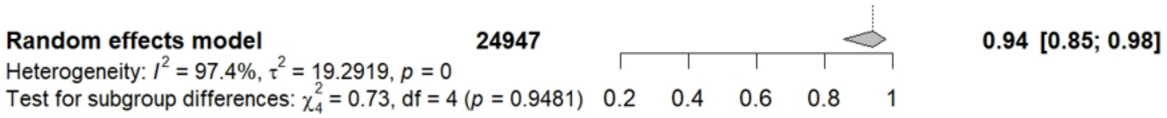

Supplementary Figure S4d. Forest Plot of Subgroup AMR Prevalence

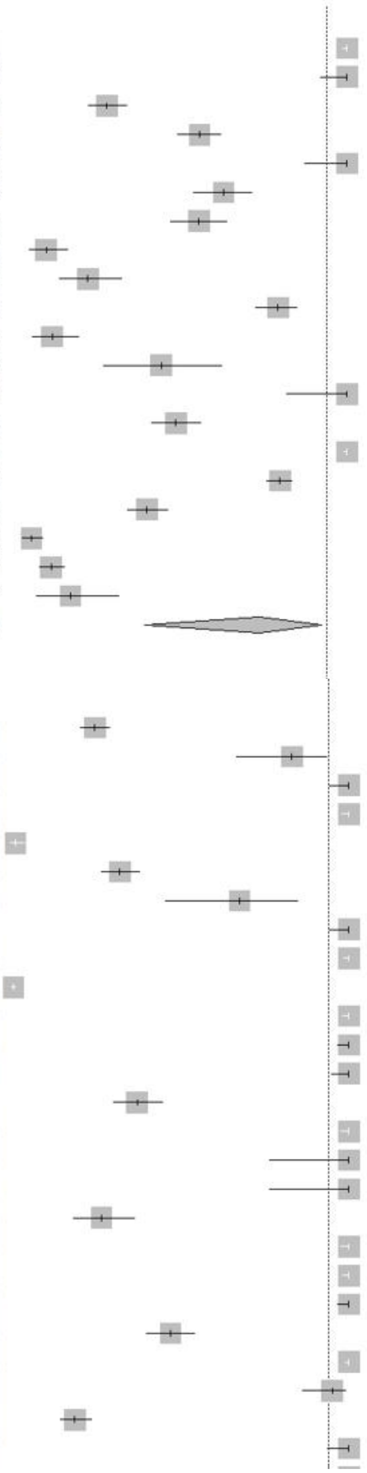

Study	Events	Total	Proportion	95%-CI
**Region = East**				
Cuypers et al. 2023	284	284	1.00	[0.99; 1.00]
Abaasa N. et al. 2024	47	47	1.00	[0.92; 1.00]
Milenkov et al. 2024	86	289	0.30	[0.25; 0.35]
Milenkov et al. 2024	140	246	0.57	[0.50; 0.63]
Milenkov et al. 2024	28	28	1.00	[0.88; 1.00]
Odoch T. et al. 2018	85	133	0.64	[0.55; 0.72]
Langata LM et al. 2019	85	150	0.57	[0.48; 0.65]
Langata LM et al. 2019	18	150	0.12	[0.07; 0.18]
Muriuki CW et al. 2022	23	95	0.24	[0.16; 0.34]
Kariuki K et al. 2023	150	188	0.80	[0.73; 0.85]
Kikuvi GM, et al. 2010	16	116	0.14	[0.08; 0.21]
Kasiano P et al. 2024	16	35	0.46	[0.29; 0.63]
Saraiva MdMS et al. 2022	19	19	1.00	[0.82; 1.00]
Negeri AA et al. 2023	100	200	0.50	[0.43; 0.57]
Ball TA et al. 2019	400	400	1.00	[0.99; 1.00]
Katakweba A.A.S. et al. 2018	390	485	0.80	[0.77; 0.84]
Eger E. et al. 2021	120	289	0.42	[0.36; 0.47]
Beyene A.M. et al. 2024	28	362	0.08	[0.05; 0.11]
Ball T. et al. 2020	51	379	0.13	[0.10; 0.17]
Ayele B. et al. 2025	9	47	0.19	[0.09; 0.33]
**Random effects model**		**3942**	**0.75**	**[0.41; 0.93]**
Heterogeneity: $I^2 = 97.3\%$, $\tau^2 = 9.8124$, $p < 0.0001$				
**Region = West**				
Aworh MK et al. 2021	110	429	0.26	[0.22; 0.30]
Adelowo O.O. et al. 2014	30	36	0.83	[0.67; 0.94]
Odih EE et al. 2022	63	63	1.00	[0.94; 1.00]
Adesoji AT, et al., 2016	168	168	1.00	[0.98; 1.00]
Touglo K. et al. 2025	7	285	0.02	[0.01; 0.05]
Touglo K. et al. 2025	94	285	0.33	[0.28; 0.39]
Rabiu AG et al. 2025	17	25	0.68	[0.46; 0.85]
Prah I et al. 2021	62	62	1.00	[0.94; 1.00]
Andrews Sah, et al. 2022	254	254	1.00	[0.99; 1.00]
Akinyemi KO et al. 2023	48	2522	0.02	[0.01; 0.03]
Leinyuy JF, et al. 2023	200	200	1.00	[0.98; 1.00]
Inwezerua C et al. 2014	114	114	1.00	[0.97; 1.00]
Ajibola A.T. et al. 2025	71	71	1.00	[0.95; 1.00]
Adesoji AT, et al., 2019	69	181	0.38	[0.31; 0.46]
Afunwa RA et al. 2011	145	145	1.00	[0.97; 1.00]
Sunmonu GT et al. 2025	14	14	1.00	[0.77; 1.00]
Sunmonu GT et al. 2025	14	14	1.00	[0.77; 1.00]
Adesoji AT et al. 2015	29	105	0.28	[0.19; 0.37]
Adenipekun EO et al. 2019	144	144	1.00	[0.97; 1.00]
Vounba P. et al. 2019	193	193	1.00	[0.98; 1.00]
Medugu N. et al. 2022	107	107	1.00	[0.97; 1.00]
Dsani E. et al. 2020	98	205	0.48	[0.41; 0.55]
Aworh M.K. et al. 2022	448	448	1.00	[0.99; 1.00]
Sunmonu G.T. et al. 2025	59	62	0.95	[0.87; 0.99]
Fashae K. et al. 2021	60	302	0.20	[0.16; 0.25]
Tettey R. et al. 2024	57	57	1.00	[0.94; 1.00]
[illegible]	[illegible]	[illegible]	[illegible]	[illegible]

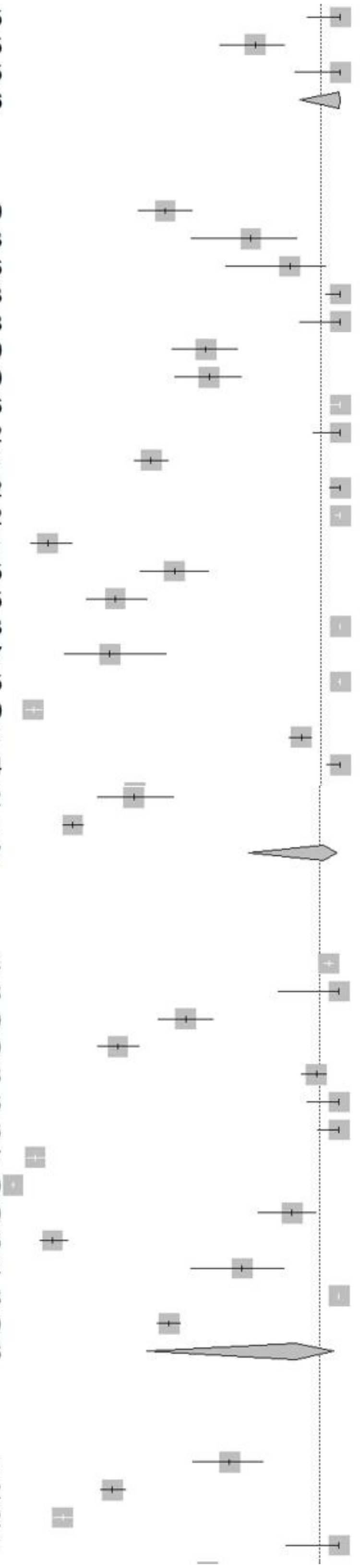

Lamikanra A. et al. 2011	35	35	1.00 [0.90; 1.00]
Adelowo O.O. et al. 2018	63	85	0.74 [0.63; 0.83]
Sow A.G. et al. 2007	25	25	1.00 [0.86; 1.00]
**Random effects model**		**6636**	**1.00 [0.88; 1.00]**
Heterogeneity: $I^2 = 95.9\%$, $\tau^2 = 35.7311$, $p < 0.0001$			
**Region = North**			
Yahiaoui M. et al. 2015	70	150	0.47 [0.38; 0.55]
Mohammed R et al. 2024	24	33	0.73 [0.54; 0.87]
Ed-Dra A et al. 2018	22	26	0.85 [0.65; 0.96]
Abbassi MS et al. 2021	83	83	1.00 [0.96; 1.00]
Barguigua A et al. 2019	28	28	1.00 [0.88; 1.00]
Messaili C, et al. 2019	59	100	0.59 [0.49; 0.69]
Soufi L et al. 2009	60	100	0.60 [0.50; 0.70]
Khalifa HO et al. 2019	126	126	1.00 [0.97; 1.00]
Al-Gallas N et al. 2021	42	42	1.00 [0.92; 1.00]
Sallem N. et al. 2024	157	371	0.42 [0.37; 0.48]
Ahmed A.M. & Shimamoto T. 2011	112	112	1.00 [0.97; 1.00]
Saidani M. et al. 2019	232	232	1.00 [0.98; 1.00]
Ben Yahia H. et al. 2018	12	111	0.11 [0.06; 0.18]
Benlabidi S. et al. 2023	47	95	0.49 [0.39; 0.60]
Hassen B. et al. 2020	33	105	0.31 [0.23; 0.41]
Hassen B. et al. 2019	2023	2023	1.00 [1.00; 1.00]
Soufi L. et al. 2012	11	37	0.30 [0.16; 0.47]
Bourjilat F. et al. 2011	535	535	1.00 [0.99; 1.00]
Salem M. et al. 2025	29	450	0.06 [0.04; 0.09]
Rebbah N. et al. 2018	327	371	0.88 [0.84; 0.91]
Aggad A. et al. 2017	84	84	1.00 [0.96; 1.00]
Ben Aissa R. & Al-Gallas N. 2008	27	72	0.38 [0.26; 0.50]
Benamrouche N. et al. 2025	117	621	0.19 [0.16; 0.22]
**Random effects model**		**5907**	**0.95 [0.72; 0.99]**
Heterogeneity: $I^2 = 96.1\%$, $\tau^2 = 19.7660$, $p < 0.0001$			
**Region = Southern**			
Mashe T et al. 2021	760	784	0.97 [0.95; 0.98]
Naidoo Y, et al. 2024	18	18	1.00 [0.81; 1.00]
Igbinosa IH 2015	80	150	0.53 [0.45; 0.62]
Estaleva CE L et al. 2021	75	230	0.33 [0.27; 0.39]
Mbelle NM et al. 2019	175	188	0.93 [0.88; 0.96]
Shawa M et al. 2021	36	36	1.00 [0.90; 1.00]
Shawa M et al. 2022	56	56	1.00 [0.94; 1.00]
Abdalla S.E. et al. 2025	31	417	0.07 [0.05; 0.10]
Takawira F.T. et al. 2022	21	3000	0.01 [0.00; 0.01]
Katsande P. et al. 2025	60	70	0.86 [0.75; 0.93]
Chirindze L.M. et al. 2018	35	275	0.13 [0.09; 0.17]
Fadare F.T. & Okoh A.I. 2021	31	44	0.70 [0.55; 0.83]
Matle I. et al. 2025	1028	1028	1.00 [1.00; 1.00]
Montso P.K., et al. 2019	376	780	0.48 [0.45; 0.52]
**Random effects model**		**7076**	**0.87 [0.41; 0.98]**
Heterogeneity: $I^2 = 98.9\%$, $\tau^2 = 15.8268$, $p < 0.0001$			
**Region = Central**			
Van Puyvelde S et al. 2019	54	81	0.67 [0.55; 0.77]
Breurec S et al. 2019	180	582	0.31 [0.27; 0.35]
Koudoum P.L. et al. 2025	103	652	0.16 [0.13; 0.19]
Irenge L.M. et al. 2019	21	21	1.00 [0.84; 1.00]

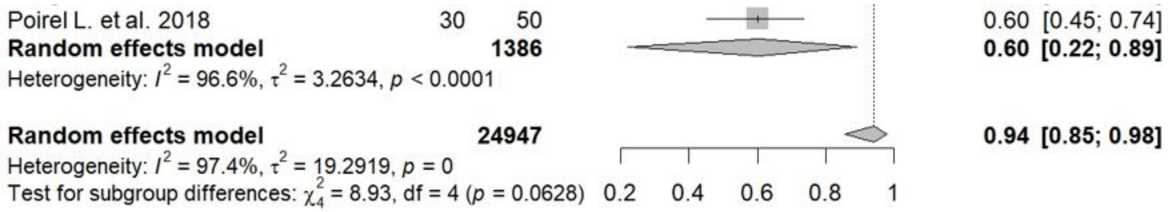

Supplementary Figure S4e. Forest Plot of Subgroup Regional Prevalence

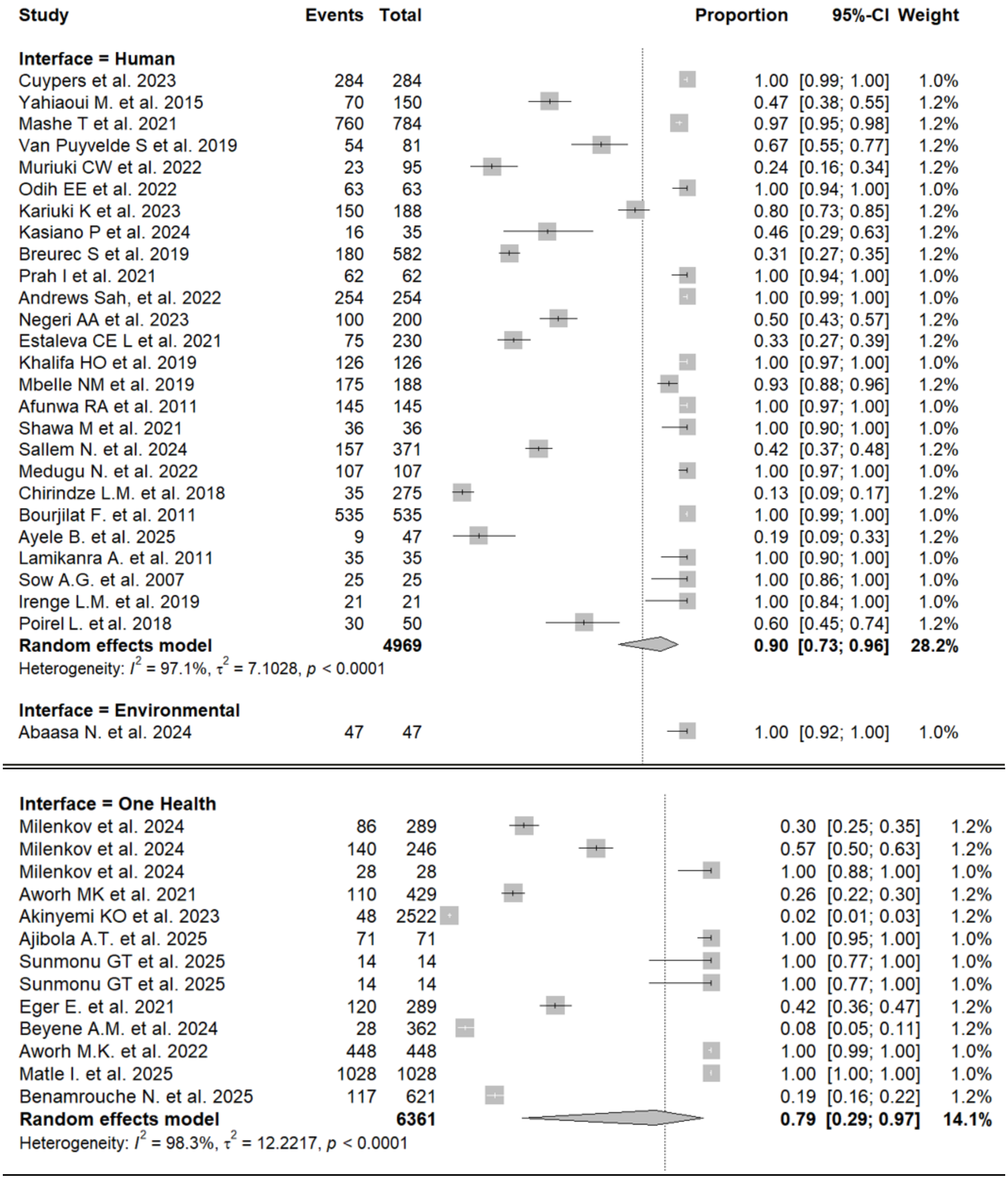
Study Events Total Proportion 95%-CI Weight
Interface = Human
Cuypers et al. 2023 284 284 1.00 [0.99; 1.00] 1.0%
Yahiaoui M. et al. 2015 70 150 0.47 [0.38; 0.55] 1.2%
Mashe T et al. 2021 760 784 0.97 [0.95; 0.98] 1.2%
Van Puyvelde S et al. 2019 54 81 0.67 [0.55; 0.77] 1.2%
Muriuki CW et al. 2022 23 95 0.24 [0.16; 0.34] 1.2%
Odih EE et al. 2022 63 63 1.00 [0.94; 1.00] 1.0%
Kariuki K et al. 2023 150 188 0.80 [0.73; 0.85] 1.2%
Kasiano P et al. 2024 16 35 0.46 [0.29; 0.63] 1.2%
Breurec S et al. 2019 180 582 0.31 [0.27; 0.35] 1.2%
Prah I et al. 2021 62 62 1.00 [0.94; 1.00] 1.0%
Andrews Sah, et al. 2022 254 254 1.00 [0.99; 1.00] 1.0%
Negeri AA et al. 2023 100 200 0.50 [0.43; 0.57] 1.2%
Estaleva CE L et al. 2021 75 230 0.33 [0.27; 0.39] 1.2%
Khalifa HO et al. 2019 126 126 1.00 [0.97; 1.00] 1.0%
Mbelle NM et al. 2019 175 188 0.93 [0.88; 0.96] 1.2%
Afunwa RA et al. 2011 145 145 1.00 [0.97; 1.00] 1.0%
Shawa M et al. 2021 36 36 1.00 [0.90; 1.00] 1.0%
Sallem N. et al. 2024 157 371 0.42 [0.37; 0.48] 1.2%
Medugu N. et al. 2022 107 107 1.00 [0.97; 1.00] 1.0%
Chirindze L.M. et al. 2018 35 275 0.13 [0.09; 0.17] 1.2%
Bourjilat F. et al. 2011 535 535 1.00 [0.99; 1.00] 1.0%
Ayele B. et al. 2025 9 47 0.19 [0.09; 0.33] 1.2%
Lamikanra A. et al. 2011 35 35 1.00 [0.90; 1.00] 1.0%
Sow A.G. et al. 2007 25 25 1.00 [0.86; 1.00] 1.0%
Irenge L.M. et al. 2019 21 21 1.00 [0.84; 1.00] 1.0%
Poirel L. et al. 2018 30 50 0.60 [0.45; 0.74] 1.2%
Random effects model 4969 0.90 [0.73; 0.96] 28.2%
Heterogeneity: I^2 = 97.1%, τ^2 = 7.1028, p < 0.0001
Interface = Environmental
Abaasa N. et al. 2024 47 47 1.00 [0.92; 1.00] 1.0%
Interface = One Health
Milenkov et al. 2024 86 289 0.30 [0.25; 0.35] 1.2%
Milenkov et al. 2024 140 246 0.57 [0.50; 0.63] 1.2%
Milenkov et al. 2024 28 28 1.00 [0.88; 1.00] 1.0%
Aworh MK et al. 2021 110 429 0.26 [0.22; 0.30] 1.2%
Akinyemi KO et al. 2023 48 2522 0.02 [0.01; 0.03] 1.2%
Ajibola A.T. et al. 2025 71 71 1.00 [0.95; 1.00] 1.0%
Sunmonu GT et al. 2025 14 14 1.00 [0.77; 1.00] 1.0%
Sunmonu GT et al. 2025 14 14 1.00 [0.77; 1.00] 1.0%
Eger E. et al. 2021 120 289 0.42 [0.36; 0.47] 1.2%
Beyene A.M. et al. 2024 28 362 0.08 [0.05; 0.11] 1.2%
Aworh M.K. et al. 2022 448 448 1.00 [0.99; 1.00] 1.0%
Matle I. et al. 2025 1028 1028 1.00 [1.00; 1.00] 1.0%
Benamrouche N. et al. 2025 117 621 0.19 [0.16; 0.22] 1.2%
Random effects model 6361 0.79 [0.29; 0.97] 14.1%
Heterogeneity: I^2 = 98.3%, τ^2 = 12.2217, p < 0.0001

Study	Events	Total	Proportion [95% CI]	Weight
**Interface = Animal**				
Odoch T. et al. 2018	85	133	0.64 [0.55; 0.72]	1.2%
Takawira F.T. et al. 2022	21	3000	0.01 [0.00; 0.01]	1.2%
Katsande P. et al. 2025	60	70	0.86 [0.75; 0.93]	1.2%
Ahmed A.M. & Shimamoto T. 2011	112	112	1.00 [0.97; 1.00]	1.0%
Saidani M. et al. 2019	232	232	1.00 [0.98; 1.00]	1.0%
Benlabidi S. et al. 2023	47	95	0.49 [0.39; 0.60]	1.2%
Hassen B. et al. 2019	2023	2023	1.00 [1.00; 1.00]	1.0%
Salem M. et al. 2025	29	450	0.06 [0.04; 0.09]	1.2%
Fashae K. et al. 2021	60	302	0.20 [0.16; 0.25]	1.2%
Montso P.K., et al. 2019	376	780	0.48 [0.45; 0.52]	1.2%
**Random effects model**		**7197**	**0.76 [0.14; 0.98]**	**11.2%**
Heterogeneity: $I^2 = 98.8\%$, $\tau^2 = 15.6464$, $p < 0.0001$				
**Interface = Animal–Environment**				
Adelowo O.O. et al. 2014	30	36	0.83 [0.67; 0.94]	1.2%
Langata LM et al. 2019	85	150	0.57 [0.48; 0.65]	1.2%
Langata LM et al. 2019	18	150	0.12 [0.07; 0.18]	1.2%
Touglo K. et al. 2025	7	285	0.02 [0.01; 0.05]	1.2%
Touglo K. et al. 2025	94	285	0.33 [0.28; 0.39]	1.2%
Abbassi MS et al. 2021	83	83	1.00 [0.96; 1.00]	1.0%
Kikuvi GM, et al. 2010	16	116	0.14 [0.08; 0.21]	1.2%
Barguigua A et al. 2019	28	28	1.00 [0.88; 1.00]	1.0%
Igbinosa IH 2015	80	150	0.53 [0.45; 0.62]	1.2%
Messaili C, et al. 2019	59	100	0.59 [0.49; 0.69]	1.2%
Saraiva MdMS et al. 2022	19	19	1.00 [0.82; 1.00]	1.0%
Leinyuy JF, et al. 2023	200	200	1.00 [0.98; 1.00]	1.0%
Ball TA et al. 2019	400	400	1.00 [0.99; 1.00]	1.0%
Soufi L et al. 2009	60	100	0.60 [0.50; 0.70]	1.2%
Abdalla S.E. et al. 2025	31	417	0.07 [0.05; 0.10]	1.2%
Vounba P. et al. 2019	193	193	1.00 [0.98; 1.00]	1.0%
Ben Yahia H. et al. 2018	12	111	0.11 [0.06; 0.18]	1.2%
Dsani E. et al. 2020	98	205	0.48 [0.41; 0.55]	1.2%
Ball T. et al. 2020	51	379	0.13 [0.10; 0.17]	1.2%
Soufi L. et al. 2012	11	37	0.30 [0.16; 0.47]	1.2%
**Random effects model**		**3444**	**0.66 [0.32; 0.89]**	**22.4%**
Heterogeneity: $I^2 = 96.6\%$, $\tau^2 = 8.3055$, $p < 0.0001$				
**Interface = Environment**				
Naidoo Y, et al. 2024	18	18	1.00 [0.81; 1.00]	1.0%
Mohammed R et al. 2024	24	33	0.73 [0.54; 0.87]	1.2%
Adesoji AT, et al., 2016	168	168	1.00 [0.98; 1.00]	1.0%
Rabiu AG et al. 2025	17	25	0.68 [0.46; 0.85]	1.2%
Adesoji AT, et al., 2019	69	181	0.38 [0.31; 0.46]	1.2%
Adesoji AT et al. 2015	29	105	0.28 [0.19; 0.37]	1.2%
Fadare F.T. & Okoh A.I. 2021	31	44	0.70 [0.55; 0.83]	1.2%
Hassen B. et al. 2020	33	105	0.31 [0.23; 0.41]	1.2%
Rebbah N. et al. 2018	327	371	0.88 [0.84; 0.91]	1.2%
Tettey R. et al. 2024	57	57	1.00 [0.94; 1.00]	1.0%
Adelowo O.O. et al. 2018	63	85	0.74 [0.63; 0.83]	1.2%
**Random effects model**		**1192**	**0.77 [0.47; 0.93]**	**12.4%**
Heterogeneity: $I^2 = 95.9\%$, $\tau^2 = 3.0779$, $p < 0.0001$				

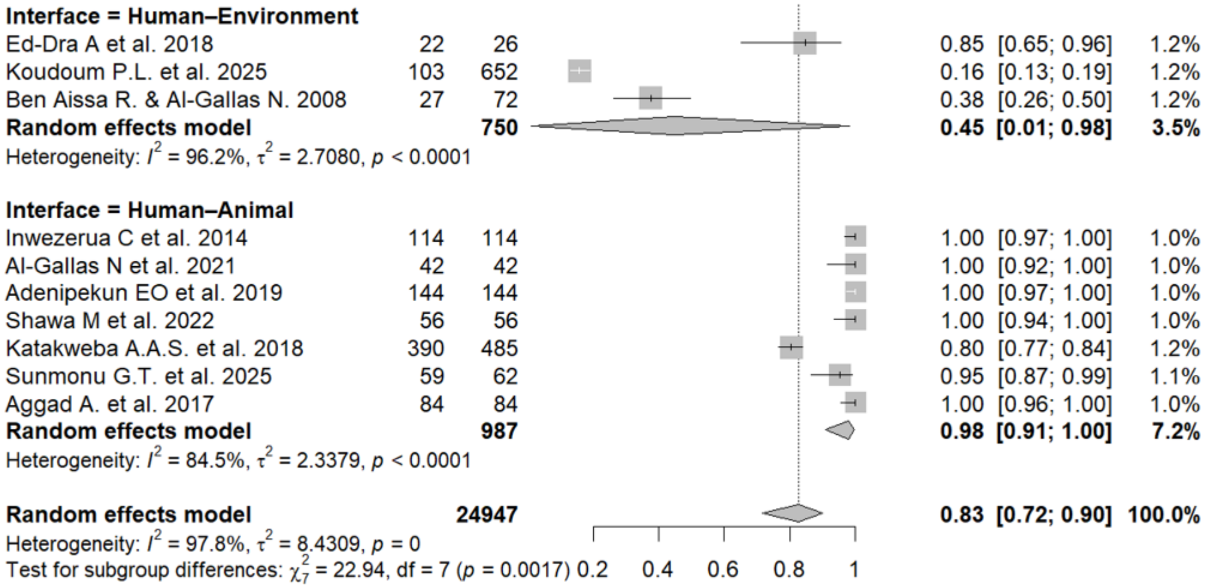

Supplementary Figure S4f. Forest plots (meta interface)

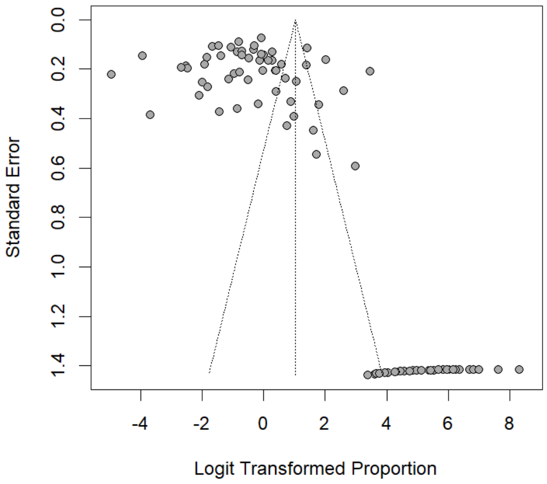

Supplementary Figure S4g. Funnel plot (publication bias)

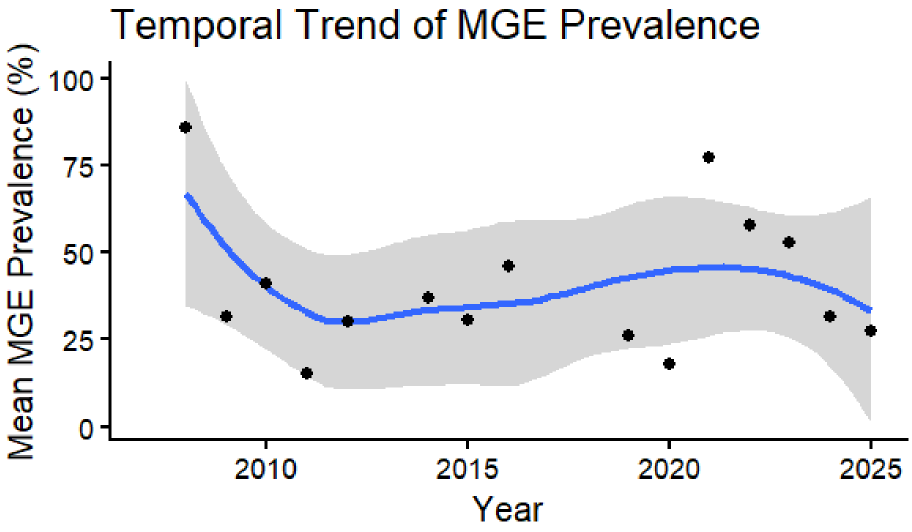

Supplementary Figure S4h. Trend of MGE Prevalence Over Time

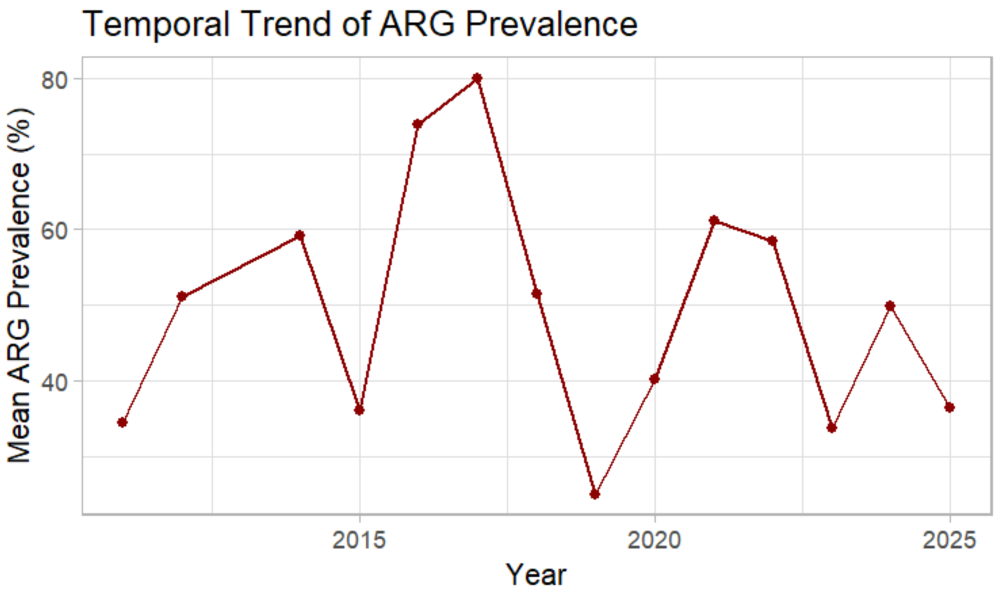

Supplementary Figure S4i. Trend of ARGs Prevalence Over Time

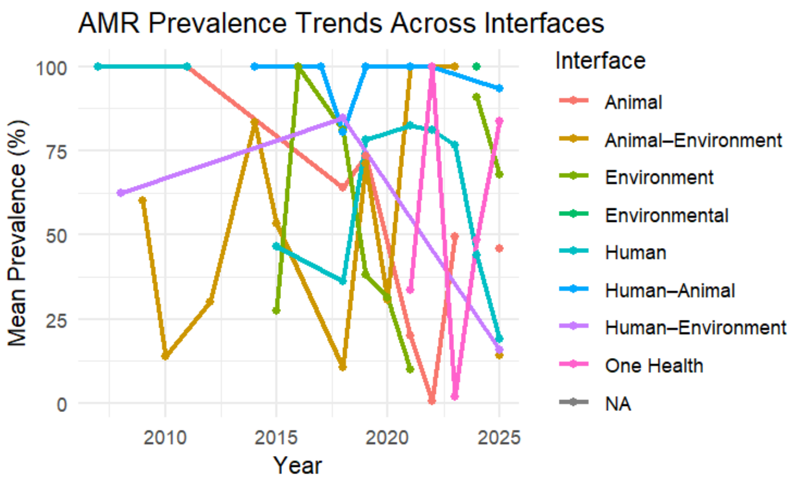

Supplementary Figure S4j. AMR Prevalence Trends Across Interfaces

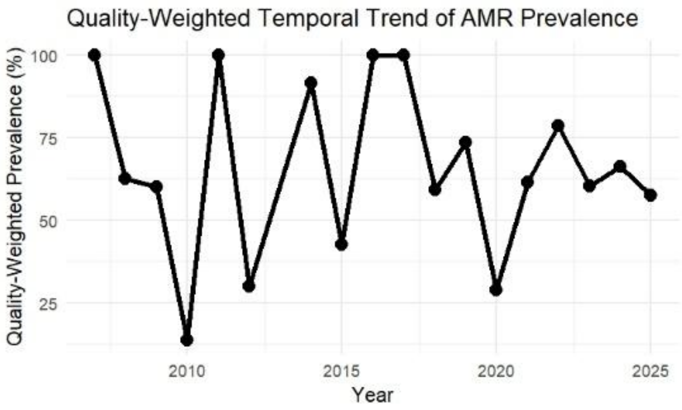

Supplementary Figure S4k. Quality-Weighted Temporal Trend of AMR Prevalence
